# Estimation of compressive strength of waste concrete utilizing fly ash/slag in concrete with interpretable approaches: optimization and graphical user interface (GUI)

**DOI:** 10.1038/s41598-024-54513-y

**Published:** 2024-02-26

**Authors:** Yakubu Dodo, Kiran Arif, Mana Alyami, Mujahid Ali, Taoufik Najeh, Yaser Gamil

**Affiliations:** 1 Architectural Engineering Department, College of Engineering, Najran University, Najran, Kingdom of Saudi Arabia; 2 Department of Computer Science, COMSATS University Islamabad, Wah Campus, Islamabad, 47040 Pakistan; 3 Department of Civil Engineering, College of Engineering, Najran University, Najran, Saudi Arabia; 4 Department of Transport Systems, Traffic Engineering and Logistics, Faculty of Transport and Aviation Engineering, Silesian University of Technology, Krasińskiego 8 Street, 40-019 Katowice, Poland; 5 Operation and Maintenance, Operation, Maintenance and Acoustics, Department of Civil, Environmental and Natural Resources Engineering, Lulea University of Technology, Luleå, Sweden; 6 Department of Civil Engineering, School of Engineering, Monash University Malaysia, Jalan Lagoon Selatan, 47500 Bandar Sunway, Selangor Malaysia

**Keywords:** Waste ingredients, Machine learning, Ensemble approaches, Statistical analysis, Permutation features importance, Civil engineering, Composites, Mechanical properties

## Abstract

Geo-polymer concrete has a significant influence on the environmental condition and thus its use in the civil industry leads to a decrease in carbon dioxide (CO_2_) emission. However, problems lie with its mixed design and casting in the field. This study utilizes supervised artificial-based machine learning algorithms (MLAs) to anticipate the mechanical characteristic of fly ash/slag-based geopolymer concrete (FASBGPC) by utilizing AdaBoost and Bagging on MLPNN to make an ensemble model with 156 data points. The data consist of GGBS (kg/m^3^), Alkaline activator (kg/m^3^), Fly ash (kg/m^3^), SP dosage (kg/m^3^), NaOH Molarity, Aggregate (kg/m^3^), Temperature (°C) and compressive strength as output parameter. Python programming is utilized in Anaconda Navigator using Spyder version 5.0 to predict the mechanical response. Statistical measures and validation of data are done by splitting the dataset into 80/20 percent and K-Fold CV is employed to check the accurateness of the model by using MAE, RMSE, and R^2^. Statistical analysis relies on errors, and tests against external indicators help determine how well models function in terms of robustness. The most important factor in compressive strength measurements is examined using permutation characteristics. The result reveals that ANN with AdaBoost is outclassed by giving maximum enhancement with R^2^ = 0.914 and shows the least error with statistical and external validations. Shapley analysis shows that GGBS, NaOH Molarity, and temperature are the most influential parameter that has significant content in making FASBGPC. Thus, ensemble methods are suitable for constructing prediction models because of their strong and reliable performance. Furthermore, the graphical user interface (GUI) is generated through the process of training a model that forecasts the desired outcome values when the corresponding inputs are provided. It streamlines the process and provides a useful tool for applying the model's abilities in the field of civil engineering.

## Introduction

**Figure 1:**
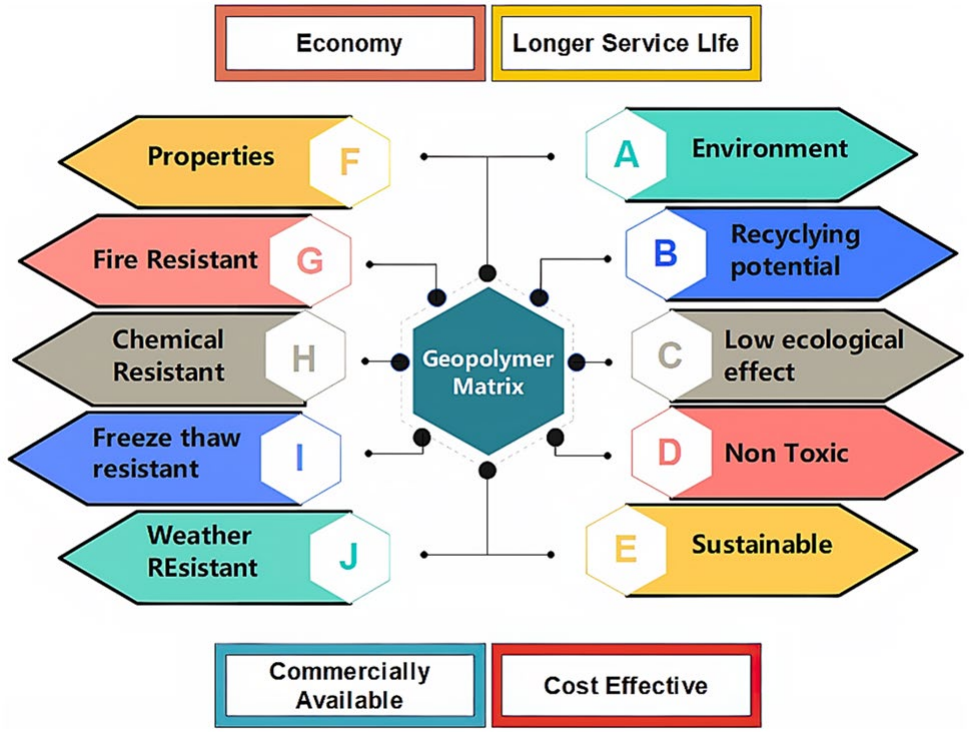
Properties of GPC.

The cement construction sector contributes significantly to global CO_2_ emissions in the construction sector^1^. The production of Portland cement (PC) emits approximately 4 billion tons of CO_2_. Thus, contributing to approximately 5–7% of total anthropogenic CO_2_ emissions in the atmosphere^2–4^. Currently, the construction industry accounts for up to 50% of global greenhouse gas (GHG) emissions^5,6^. Literature reveals that around 4000 million tons of cement are manufactured globally and will continue to rise to 6000 million tons by 2060^6^. This increase in demand for cement will have a malignant effect on the environment. Therefore, initiative and effort are needed to minimize the building industry's reliance on cement. The use of waste or recycled material including supplementary cementitious materials (SCMs), such as volcanic ash (VA)^7^, recycled aggregate (RA)^8^, fly ash (FA)^9^, ground granulated blast furnace slag (GGBS)^10^, and limestone powder (LP)^11^ in concrete production can aid in alleviating the adverse impacts on environment^12–14^. This will not only fulfill the increasing need for cement in the building sector, but it will also minimize future environmental risks^15–17^. SCMs are often utilized in the construction sector as a partial substitute for cement to reduce the environmental effect of cement manufacture and usage in concrete^18–20^. Moreover, it is reported that using natural pozzolanic materials having a rich concentration of silica and alumina such as FA and GGBS in concrete reduces the GHG emissions by 80% rather than in conventional concrete^21^. In addition, alkali-activated materials (AAM) have recently been utilized in combination with industrial materials and alkaline activators such as NaOH and Na_2_SiO_3_ to create geopolymer concrete (GPC)^22–24^. The amorphous gel-like structure of GPC provides remarkable and attractive properties including sulfate attack, resistance to acid^25^, better durability^26^, resistance to fire^27^, and obstinately higher compressive strength^28^, as shown in Fig. 1. The incorporation of FA and GGBS in making a gel-like structure making a geo-polymerization compound can give the utmost benefit in the cementitious composite matrix^10^. Thus, its incorporation in matrix not only gives desirable properties but will substantially reduce CO_2_ emission by up to 25–45%^29^.

## Alkali activated-based geopolymer concrete (AA-GPC)

Fly ash (FA) is a waste product of the thermal coal manufacturing process. It is pozzolanic that have the properties of binding materials which get polymerized under high temperature with an alkaline medium to make GPC. As a result, a crystalline and amorphous combination is created, which may then be used to provide the necessary mechanical characteristics. Due to the significant requirement for heat curing, however, field applications of geo-polymerization chemicals are not advised^30,31^. Due of its high curing temperature requirement, FA-GPC application in the construction sector will be restricted^31^. Therefore, by using a slag combination with a high concentration of calcium, silica, and alumina, heat demand may be decreased^32^. When GGBS and FA are combined, calcium aluminosilicate hydrate (C-A-S-H) is created as a byproduct.This gel is responsible for making a dense gel structure in GPC. Thus, its utilization can achieve early strength and produces enhanced mechanical properties^33^. Mehta et al.^34^ studied the mechanical properties of FA-GPC with GGBS by varying its concentration. The author observed a compact microstructure containing hydration and polymerization products, which greatly improved the strength of GPC during its early stages. Yazdi et al.^35^ examined the impact of GPC by altering the FA concentration from 30 to 100% in combination with GGBS. The author illustrates that substituting 50% of fine aggregate (FA) with ground granulated blast furnace slag (GGBS) leads to a significant enhancement in both compressive and flexural strength, with values of 100 MPa and 10 Mpa correspondingly. In addition, Fang et al.^31^ investigated the impact of slag on the flexural and split tensile strength of FA-GPC and found that it resulted in increased strength levels. This phenomenon occurs as a result of the creation of C-A-S-H gel and N-A-S-H gel by the manipulation of GGBS concentration, leading to an expedited reaction process^36^. Moreover, Table 1 illustrates the mixing regime of FA-GGBS-based GPC. Hence, the utilization of blend slag with FA in making GPC results in the decrease of CO_2_ emission in the atmosphere, disposal of waste, and consumption of energy to make eco-friendly concrete.

**Table 1:** Mix regime of FA/GGBS-based GPC.

S. no	FA (kg/m^3^)	GGBS (kg/m^3^)	Aggregates		Alkaline activator		NaOH molarity (M)	Water (kg/m^3^)	Curing temperature (°C )	Strength (MPa)	References
			Coarse (kg/m^3^)	Fine (kg/m^3^)	NaOH (kg/m^3^)	Na_2_SiO_3_ (kg/m^3^)					
1	0	500	1115	600	50	125	12	24	24	59.5	^37^
2	50	450	1115	600	50	125	12	24	24	59.0	
3	100	400	1115	600	50	125	12	24	24	58.2	
4	150	350	1115	600	50	125	12	24	24	49.2	
5	200	300	1115	600	50	125	12	24	24	42.5	
6	250	250	1115	600	50	125	12	24	24	40.9	
7	300	200	1115	600	50	125	12	24	24	35.9	
8	349.2	38.8	1221.2	620.8	194.0		12	13.3	23	41.7	^38^
9	349.2	38.8	1221.2	620.8	194.0		12	13.3	60	50.0	
10	349.2	38.8	1221.2	620.8	194.0		12	13.3	75	62.3	
11	349.2	38.8	1221.2	620.8	194.0		12	13.3	90	60.7	
12	400	0	1209	651	45.7	114.3	14	22	22	26.7	
13	360	40	1209	651	45.7	114.3	14	22	22	34.6	^39^
14	320	80	1209	651	45.7	114.3	14	22	22	45.6	
15	280	120	1209	651	45.7	114.3	14	22	22	54.8	
16	225	225	1164	627	45	112.5	14	24	24	41.1	^40^
17	200	200	1068	712	12	74		24	24	50.0	
18	300	100	1068	712	12	74		24	24	51.3	^28^
19	400	0	1068	712	12	74		24	24	63.4	

An experimental mix design is utilized to establish the optimum loading capacity of concrete. However, prior research has shown that GP concrete is significantly influenced by the chemical composition and physical amounts of factors. There exists heterogeneity in making GP concrete due to its diverse factor and still their exit ambiguity in making a mixture^41–43^. However, many methods and methodologies based on statistical approaches may be used to analyze the crushing nature of GPC. Despite the little study into the link between variables and mechanical properties. Recently, the use of machine learning algorithms (MLAs) and their application in concrete mix design has increased in the last decades^44–48^. This is due to its non-linearity relationship and its complex behavior that takes the uncertainty and predicts the most influential results by taking dependent and independent variables. MLAs frequently split the data samples into the training set and testing set. The employed algorithms in making a model trains it by using a training set and predicting the outcome on the test set. The usefulness of MLAs is often lying in their nonlinearity relation due to their automatically complex iteration to achieve accurate prediction^49–51^. Furthermore, empirical models, iterate once in the data sets and predict the outcome with fitted variables that is why linear regression is not useful in getting accurate and desirable predictions.

**Figure 2:**
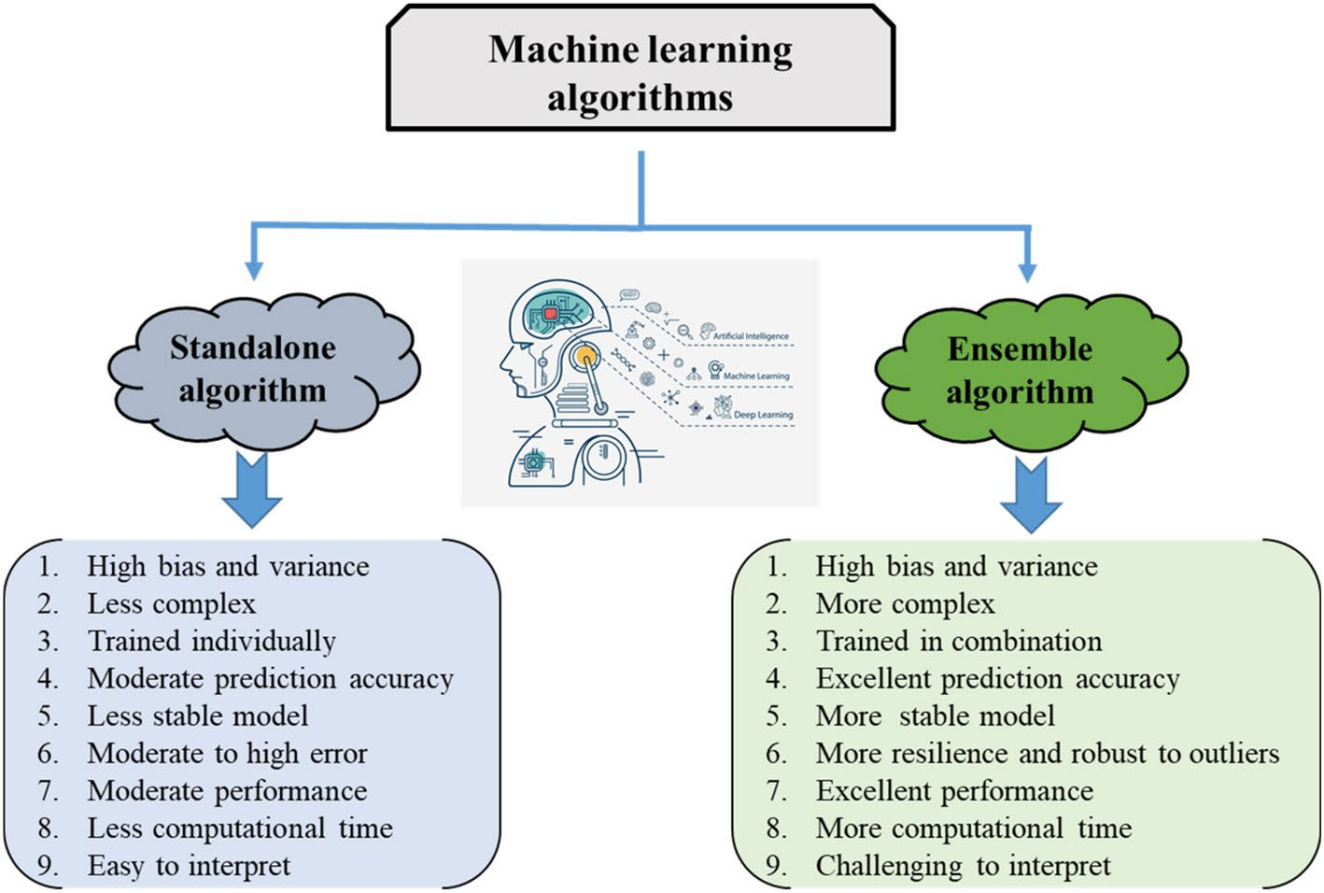
Difference between individual and ensemble approaches.

Lokuge et al.^41^ predict the compressive nature of FA-GPC by employing the Multi-variate Adaptive Regression Splines (MARS) algorithm. The author predicts robust performance by using nonlinear regression. Mohsin et al.^52^ employed two approaches namely as random forest and gene expression programming by using 298 data points, and the results showed that RFR provides a more accurate performance than GEP with R = 0.9826. Similarly, Ayaz et al.^53^ predict the loading capacity of FA-GPC on 154 data samples that was gathered from literature by employing supervised machine learning approaches. The author achieved a good correlation of R^2^ = 0.97 by using the bagging algorithm as compared to AdaBoost and decision tree algorithms. Unluer et al.^54^ used different MLAs including support vector machines (SVM), backpropagation neural networks (BPNN), and extreme learning machines (ELMs) for the prediction of FA-GPC with 110 data points. The author concluded that SVM outclasses ELM and BPNN with a higher correlation for testing set with R^2^ = 0.955. Ali et al.^55^ utilized 399 data sets to estimate the compressive nature of GPC using an artificial neural network (ANN) by using two hidden layers of ANN. The author obtained an excellent response with R^2^ = 0.9916 as compared to other layers. In addition, Mohsin et al.^42^ forecast the mechanical characteristics of GPC using the GEP technique. The results show a significant connection with the empirical equation. Similarly, Alkaroosh et al.^56^ forecast the GPC strength by utilizing the GEP model with 56 data points and reported a good correlation with R^2^ = 0.89 for validation set. Aneja et al.^57^ forecasted the compressive strength of FA and BA-based GPC by using artificial neuron network by gathering 46 data points from the literature and made eighteen samples by conducting experimental work. Fourteen ANN models that differ in hidden layers, backpropagation training algorithms, and neuron was tilized. Bayesian Regularization (BR), Levenberg–Marquardt (LM), and scaled conjugate gradient (SCG) as backpropagation algorithms was employed. Moreover, number of neurons, hidden layers and training approaches have siginificant influence of ANN models. Moreover, model evaluation is gauged by mean square error (MSE), and coefficient of correlation ®. The author reveals that increasing hidden layers decreases the MSE and increases the R value of BR and LM models. In addition, eight model (ANN-VIII) BR-ANN with three layers and 10 neurons demonstrates accurate prediction with R = 0.99 and lesser MSE = 1.017. Dong et al.^58^ forcasted the compressive strength of GPC with artificial neural network and adaptive neuro fuzzy inference (ANFIS). The author use four input parameters for prediction and reported that ANFIS performs much better then ANN in term of R^2^ and statistical measures. Cao et al.^59^ utilized three approaches namely as support vector machine (SVM), extreme gradient boosting (XGB), and multilayer perceptron (MLP) for predicting the compressive strength of GPC. The author reveals that XGB approach outclass by depict higher correlation R^2^ = 0.98 as compared to SVM and MLP which is 0.91 and 0.88, respectively. In addition, statistical measures and validation with K-fold confirms the high accuracy of XGB. Simlarly, Ashrafian et al.^60^ anticipated the apparent chloride concentration of marine structure having different zones. The author employees gene expression programming (GEP), Multivariate Adaptive Regression Splines (MARS), and M5p Model Tree (MT) on 642 data points, and observed that MARS outperformed outclass from both approaches. In addition, Ashrafian et al.^61^ predicted the slump flow of self compacting concrete (SCC) with 117 data points by utlizining MARS and MT approach, and observed both models have greater potential with higher precision. Chu et al.^62^ anticipated the mechanical strength of FA-GPC on 311 data points by employing GEP and MEP techniques and revealed that GEP is far superior to MEP as it gives an empirical relation with accuracy. Ashrafian et al.^63^ anticapted the strength of waste concrete by employing various machine learning approaches and demonstrated that fuzzy model with HOA optimizer depicts robust performace as compared to remaining models. Thus, MLAs have been used in the civil engineering domain due to their extraordinary response to prediction as listed in Table 2. MLA employs statistical analysis and database approaches to extract correlations, undiscovered patterns, and data from massive datasets. Prediction and modeling often use one of the two approaches. The first option, the traditional technique, is based on a stand-alone model^64^. The second approach makes use of several ensemble learning algorithms including bagging, boosting, and random forests. The newly developed ensemble learning algorithms significantly outperformed traditional ML models when predicting outcomes^65–67^. The training data is used to educate a large number of weak learners, and then those learners are combined to produce strong learners using the ensemble learning approach. These weak learners are developed using individualized learning techniques such as ANN, SVM, and DT. These recently established ensemble techniques may be used to investigate the properties and resilience of complex materials, such as HPC made from waste. Ensemble learning and classifier generation algorithms have been the subject of recent studies because of their potential to improve machine learning model performance. Likewise, Ahmad et al.^68^ utlizes adaBoost, decision tree and random forest approaches with 207 samples to predict the high-temperature compressive strength of concrete. The author observed that AdaBoost approach give strong R^2^ = 0.938 as compared to other approaches, and cement concentration (CC) of the mixture was the most sensitive variable in prediction. Chou et al.^69^ used MLP, SVM, a classification and regression tree (CART), and linear regression (LR) to develop separate and combined classifiers for learning. The results demonstrated that HPC (high-performance concrete) compressive strength may better predicted using ensemble learning approaches than using individual learning methods. To foretell HPC’s compressive and tensile strengths, Nguyen et al.^69^ used prediction algorithms such SVM, MLP, GBR, and XGBoost. The author demonstrated that GBR and XGBoost performed better as compared to SVM and MLP. The distinction between standalone algorithms and ensemble methods is also shown in Fig. 2. It also shows how individual algorithms and ensemble techniques differ from one another.

**Table 2:** Forecasting of mechanical properties by using MLAs.

Sr. no	Methods employed	Symbolization	Data points	Forecasted properties	Year	Material used	References
1	Individual and ensemble algorithm	GEP, DT and Bagging	270	Crushing strength	2021	FA	^70^
2	Data envelopment analysis	DEA	114	Compressive strength Slump test L-box test V-funnel test	2021	FA	^71^
3	Individual algorithms	ANN, GEP, DT	642	Chloride Concentration	2021	FA	^72^
4	Individual using ensemble modeling	ANN, bagging and boosting	1030	Crushing strength	2021	FA	^73^
5	Support vector machine	SVM	115	Slump test L-box test V-funnel test Crushing strength	2020	FA	^74^
8	Support vector machine	SVM	–	Crushing strength	2020	FA	^75^
9	Neuro-fuzzy inference system that is adaptable	ANFIS with ANN	7	Crushing strength	2020	POFA	^76^
10	Random forest and gene expression programming	RF and GEP	357	Crushing strength	2020	–	^77^
11	Gene expression programming	GEP	357	Crushing strength	2020	–	^78^
12	Fuzzy rules and intelligent rule-based improved multiclass support vector machine	IREMSVM-FR with RSM	114	Crushing strength	2019	FA	^79^
13	Artificial neuron network	ANN	205	Crushing strength	2019	FA GGBFS SF RHA	^80^
14	Multivariate adaptive regression spline	M5 MARS	114	Crushing strength Slump test L-box test	2018	FA	^81^
21	Artificial neuron network	ANN	300	Crushing strength	2009	FA	^82^
22	System of adaptive neurofuzzy inference	ANFIS	55	Compressive strength	2018	–	^83^

Studies reveal that nonlinear regression is used to obtain the optimal performance of low calcium FA-GPC but no nonlinear regression MLAs by employing fly ash slag-based GPC. In this research work, AdaBoost and bagging on MLPNN were used to make an ensemble model to predict the mechanical strength of mixed GPC by conducting a literature review in an anaconda navigator using Sypder version 5.1.5. A vast data set of about 156 is gathered from the literature and Web of Science present in supplementary file S1. Additionally, K-Fold cross-validations, statistical analysis, and external validation checks are used to assess the correctness of the models. Shapley analysis is done to give the most influential variable. Furthermore, a graphical user interface (GUI) is made for practical implementation in the industry for concrete prediction. To the best author’s knowledge, no work is reported by conducting nonlinear regression by employing AdaBoost and bagging on FASBGPC.

## Data description

**Figure 3:**
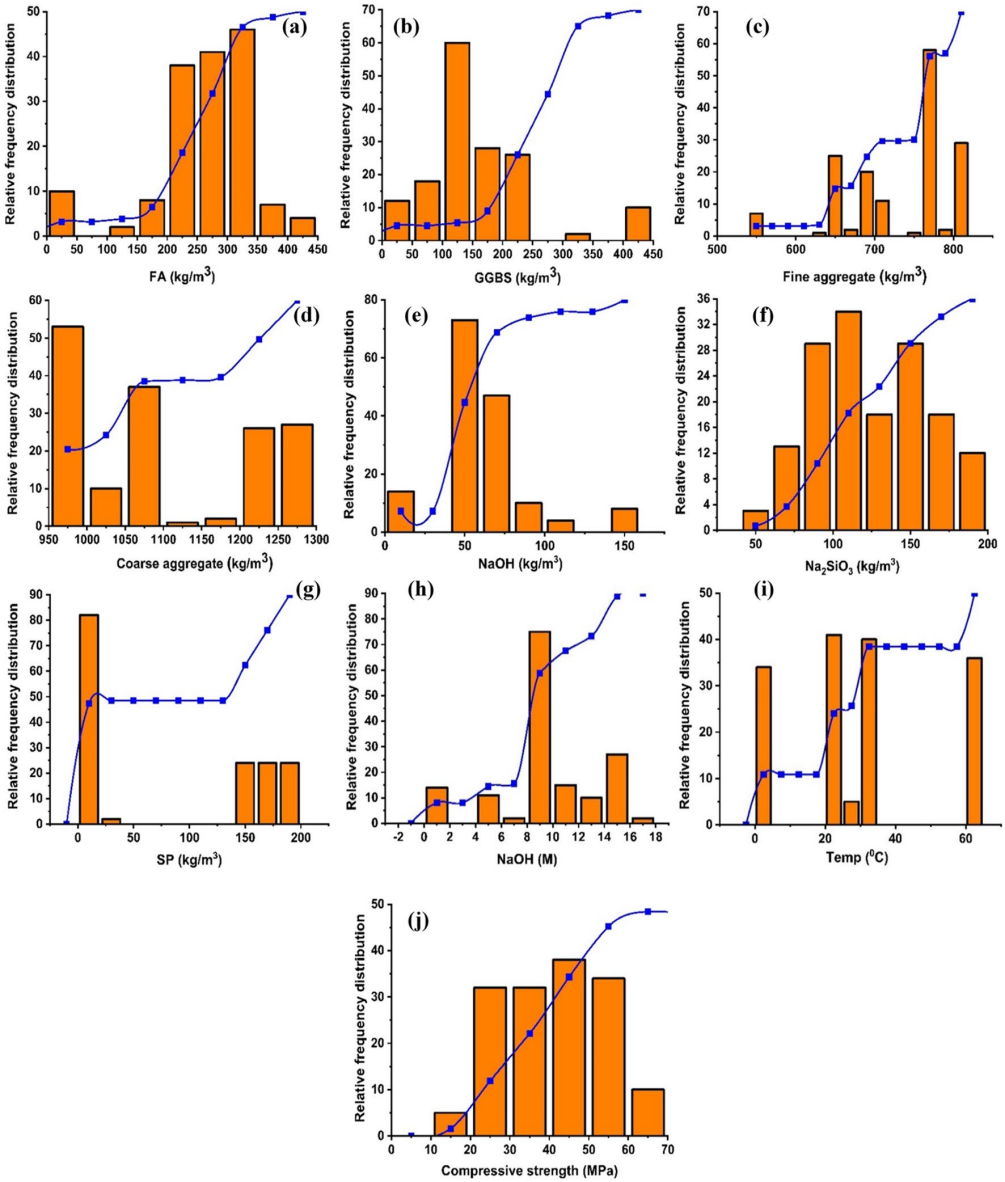
Parameters used in making an ensemble model; (**a**) fly ash (kg/m^3^), (**b**) granulated blast furnace slag (kg/m^3^), (**c**) fine aggregate (kg/m^3^), (**d**) coarse aggregate (kg/m^3^), (**e**) NaOH (kg/m^3^), (**f**) Na_2_SiO_3_, (**g**) super plasticizer (kg/m^3^), (**h**) sodium hydroxide (M), (**i**) temperature (°C), (**j**) compressive strength (MPa).

**Figure 4:**
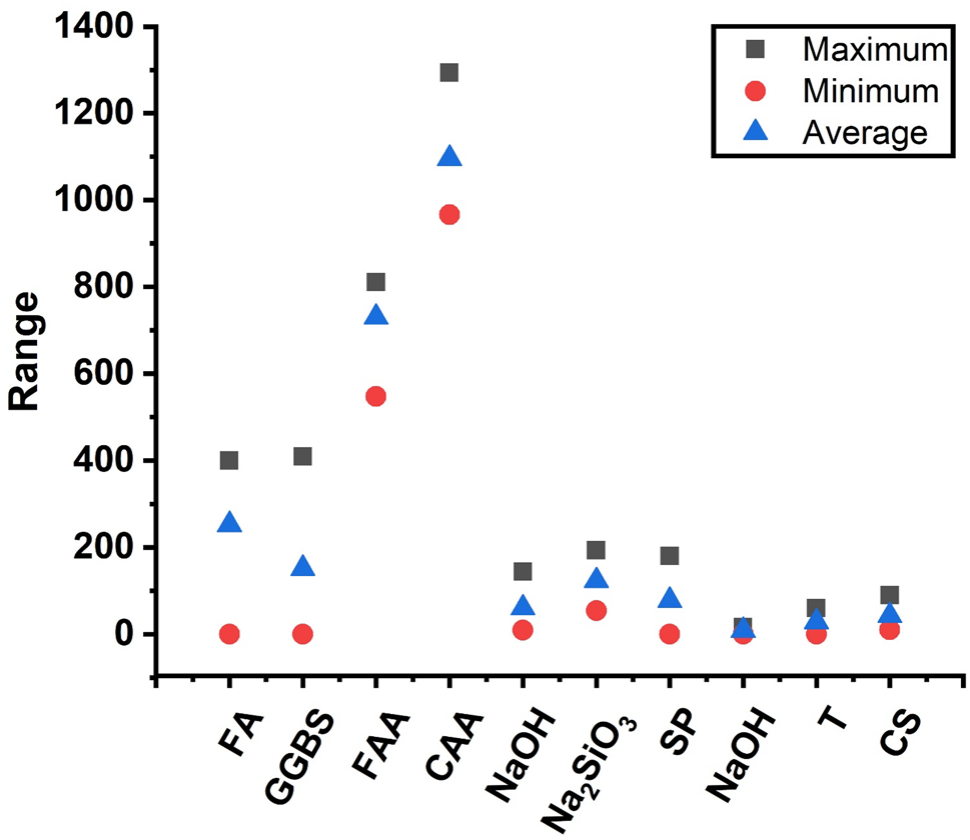
Parameter maximum and minimum value.

The data of GP concrete containing FA and GGBS has been taken from the published literature to model the strength. Furthermore, the effectiveness of the gathered data is entirely dependent upon the data points and the variable used to make a model (see supplementary file S1). The parameters used to make model compromises of fly ash (kg/m^3^), granulated blast furnace slag (kg/m^3^), fine and coarse aggregate (kg/m^3^), alkaline activators (kg/m^3^), superplasticizer (kg/m^3^), sodium hydroxide (M), temperature (°C). Moreover, the descriptive statics with frequency distribution is shown in Table 3 and Fig. 3. In contrast, the maximum and minimum values with their averages are also shown in Fig. 4.

**Table 3:** Parameter descriptive statistics.

Statistical description	FA	GGBS	Fine	Coarse	NaOH	Na_2_SiO_3_	SP	NaOH	Temp
Mean	252.5	151.4	729.8	1096.0	60.5	123.0	77.6	8.6	28.1
Standard error	6.9	6.9	5.4	9.4	2.1	2.9	6.5	0.3	1.6
Median	270.0	135.0	760.5	1090.8	57.1	115.7	7.9	8.0	25.0
Mode	303.8	101.3	774.0	1090.8	81.0	81.0	0.0	8.0	30.0
Standard deviation	86.3	86.7	68.0	117.9	26.8	35.7	81.0	3.9	20.6
Sample variance	7442.7	7522.7	4620.5	13,889.3	720.4	1275.1	6558.3	15.2	422.4
Kurtosis	2.5	2.2	0.0	− 1.5	3.0	− 0.9	− 1.9	0.2	− 0.9
Skewness	− 1.4	1.3	− 0.8	0.3	1.2	0.1	0.2	− 0.5	0.3
Range	400.0	409.0	263.6	327.0	134.3	138.9	180.0	16.0	60.0
Minimum	0.0	0.0	547.0	966.0	9.0	54.0	0.0	0.0	0.0
Maximum	400.0	409.0	810.6	1293.0	143.3	192.9	180.0	16.0	60.0
Sum	39,384.5	23,624.5	113,849.2	170,980.4	9432.3	19,185.4	12,100.6	1336.0	4380.0
Count	156.0	156.0	156.0	156.0	156.0	156.0	156.0	156.0	156.0

**Figure 5:**
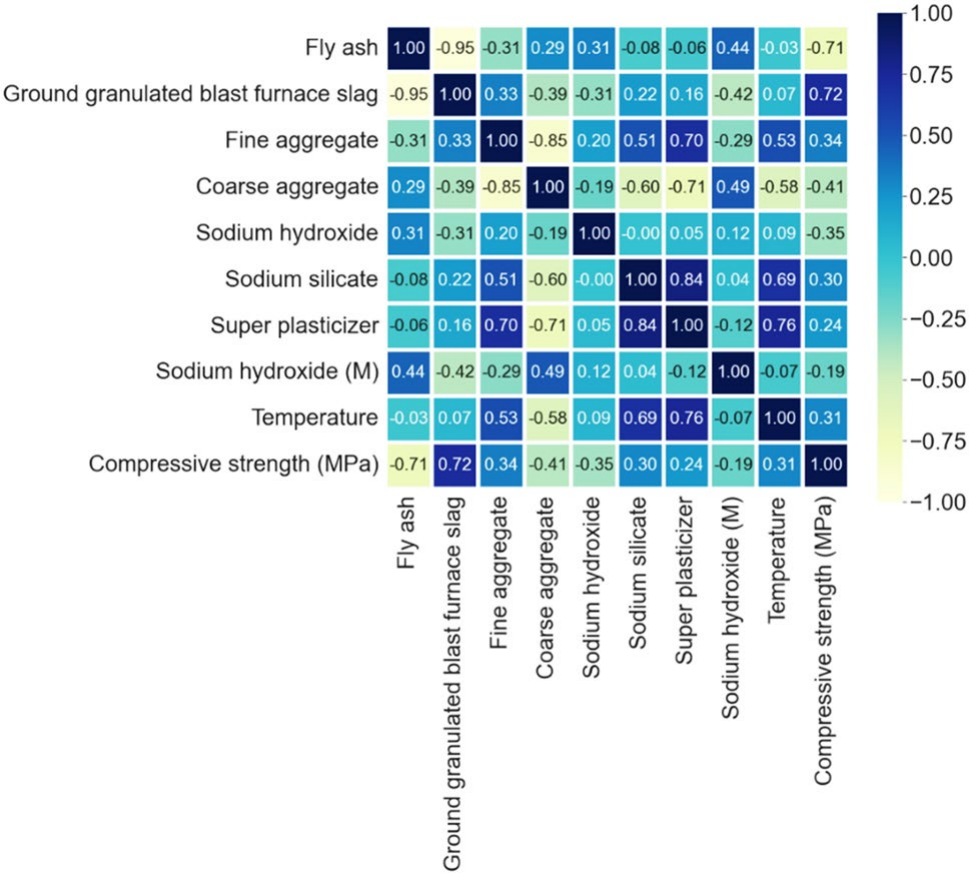
Pearson correlation of input parameters to output strength.

Moreover, the pearson correlation coefficient shows the linear relationship between input and output variables,ranging from -1 to 1 as illustrated in Fig. 5. In general it can be depicted that the correlation between inputs and outputs are weak, varying from [− 0.03 − 1]. In addition the strongest correlation between ground granulated blast furnace slag and flyash. Consequently, each of the nine inputs to the dataset can be considered independent, enabling each variable to be used to construct machine learning models and conduct feature significance and sensitivity analyses.

It is evident that the Flyash/slag based material has a significant role in enhancing strength. The reason for this is the Pozzolanic activity of both fly ash and GGBFS. Pozzolanic materials have the ability to react with calcium hydroxide, which is produced during cement hydration, when water is present. This reaction leads to the formation of more cementitious compounds. The pozzolanic reaction generates calcium silicate hydrates (C-S-H) and other cementitious substances, which efficiently occupy the empty spaces and improve the overall strength and density of the concrete structure. Moreover, their addition will reduces the water content, filler effect, and enhanced hydration reaction that ultimately increase in strength when fly ash and GGBFS are added to concrete mixtures, making them valuable and sustainable additions to enhance concrete performance.

## Methodology

**Figure 6:**
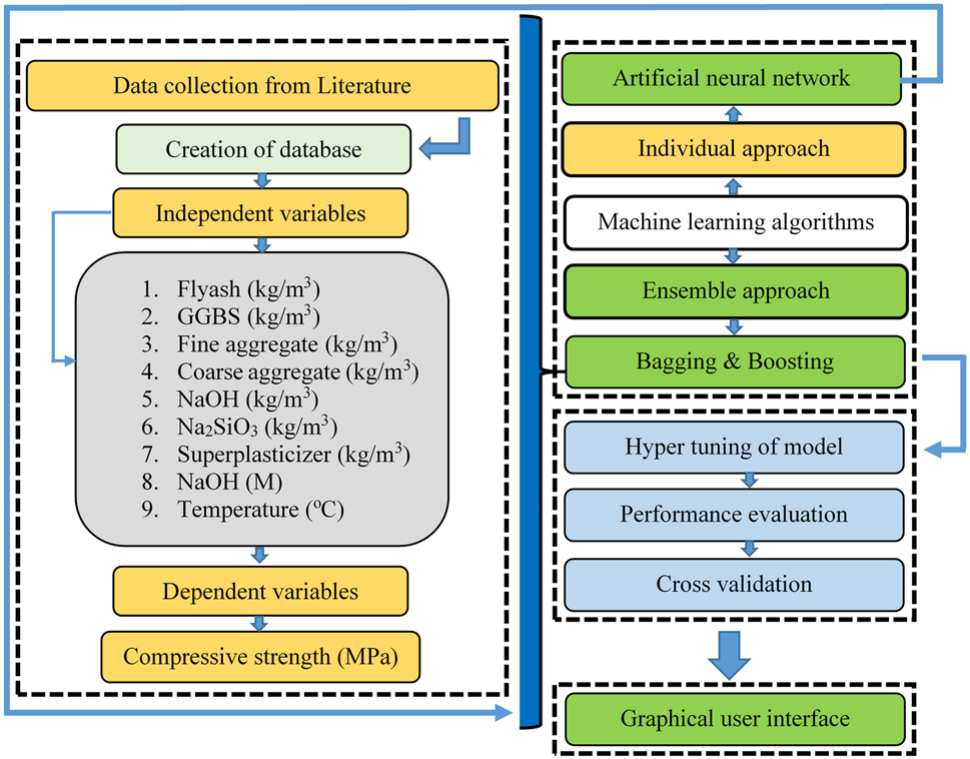
Flowchart of current research.

The data description is used to create a models to predict the compressive property of FASBGPC, statistical analysis, parametric study of the influencing variable, and the algorithms used to create the model. This sections discuss the effect of all these in details in making predictive models. Moreover, Sklearn library in Python is employed for interpretability analysis of models, which was trained by using an Artificial Neural Network (ANN) architecture. The ANN was chosen due to its effectiveness in capturing complex patterns in the data and its flexibility in handling various types of input features. In addition, hyperparameter tuning on the ANN model is used to optimize its performance to find the best performance. In addition, ensemble techniques, specifically bagging and boosting is performed to enhance the predictive performance of the ANN model. Parameter tuning was conducted not only for the individual ANN model but also for the ensemble approaches to fine-tune their hyperparameters for improved performance. Moreover, Fig. 6 illustrates the overall flowchart used in this research.

### Multilayer perceptron neuron network (MLPNN)

**Figure 7:**
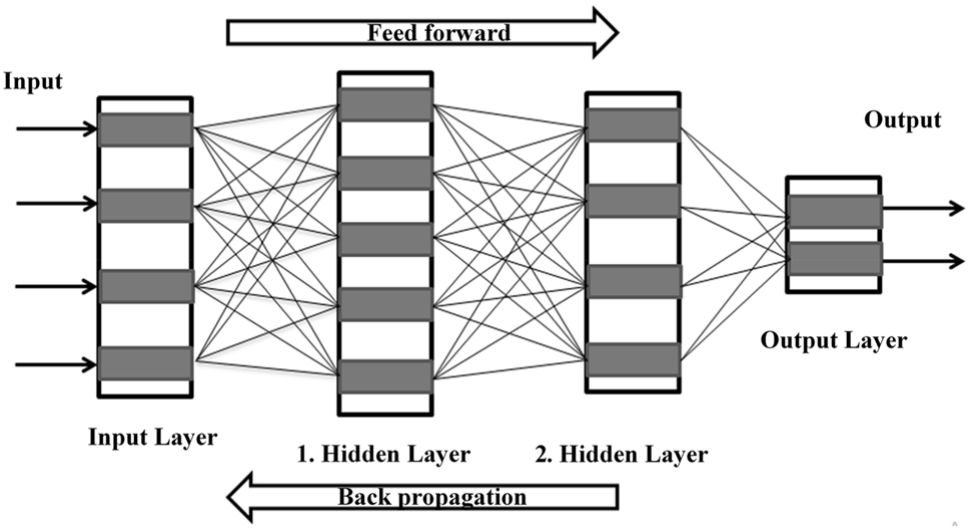
ANN-based feed-forward process.

Biological nervous system microstructure (neurons) is simulated by artificial neural network (ANN) algorithms. ANN networks are made up of a lot of parallel connections. These cells receive biased contributions from ANN neurons and then provide the biased production to other neurons through an stimulation function. There are one or more multilayers in these neutral activities. Moreover, ANNs uses neural activities as a path between network. The perception response is determined by the numeral input variables, output, and input layers. Individually network of ANN is made up of three standard layers namely the input layer, output layer, and hidden layer. The hidden layer which acts as a source of accuracy by doing a nonlinear performance on the parameters using activation function is located between the input layer and the output layer. Moreover, the hidden layer can be one or more depending on the accuracy of the model. Although a single hidden layer can handle all of the challenges that a perceptron encounters, it is more beneficial and efficient to employ multiple hidden layers. Figure 7 shows the architecture of a neural network, which contains an input layer, two hidden layers, and an output layer. Except for the input layer, every neuron in a layer determines the linear combination and bias. The neutrons then utilize the model’s output to compute the non-linear activation function sigmoid in their input.

This research study performs ANNs modeling with the use of a multi-layer perceptron (MLPNN) feed-forward network. The best performance of the model is evaluated by changing the numbers of neurons and hidden layers. Afterward, the gathered data from the literature is split into testing and training sets. This is done by splitting the data into random sets into a ratio of 80% (train set) and 20% (test set) to mitigate the overfitting effect and bias of the model in predicting the mechanical property of FASBGPC.

### Hyper-tuning parameters

**Figure 8:**
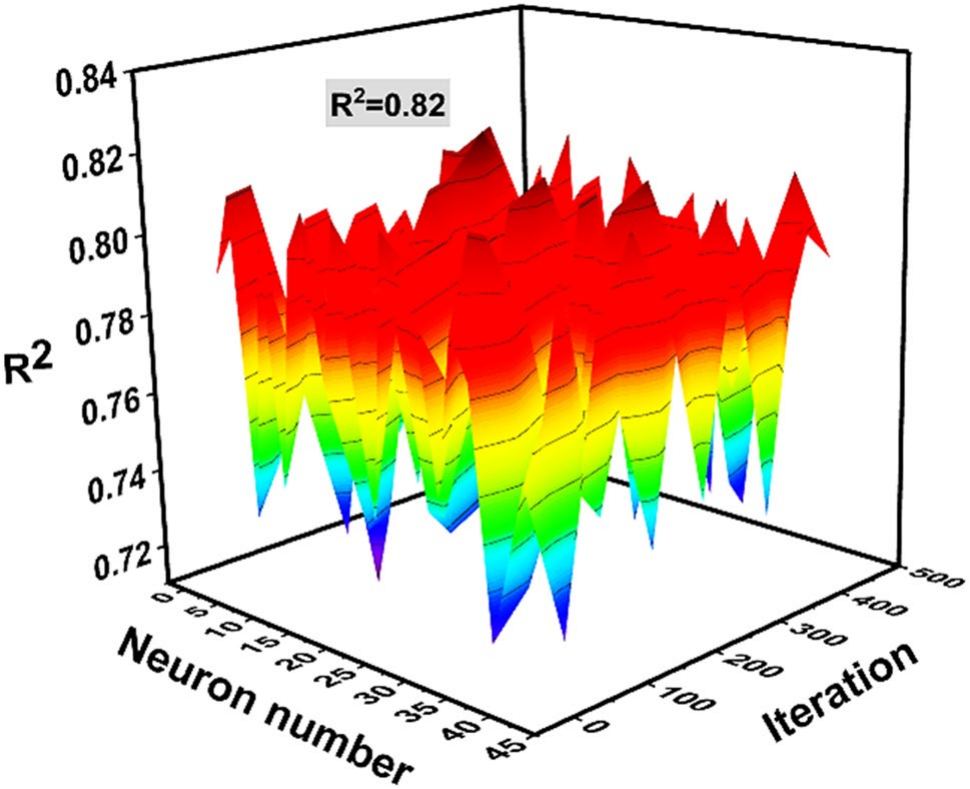
Optimization of the model by hit and trial method to achieve the best performance.

The selection of appropriate parameters are crucial in making ML models to obtain the highest accuracy. In this study, numerous hyper-parameter combinations were investigated to improve the model’s accuracy, as shown in Table 4. In addition, Fig. 8 demonstrates the ANN model with optimal parameters. Furthermore, a critical stage in the development of non-linear models is selecting the appropriate hyper-parameters. As a result, testing multiple configurations to discover the one that would operate correctly and stably when constructing ML models may require a significant amount of effort. The details of parameters selection is can also be seen in supplementary file S1.

**Table 4:** Model learning with parameters tuning.

Method utilized	Hyper parameters		
	Title	Values considered	Optimal
Artificial neural network	Hidden layer	2–40	14
	Max iter	0–450	250
	Solver	Sgd, lbfgs, adam	Adam
	Activation	Identity, logistic, tanh, relu	Relu
	Learning rate init	0.01, 0.05, 0.1, 0.15, 0.2	0.1
	Learning rate	Constant, invscaling, adaptive	Constant

### Ensemble methods using boosting and bagging approach

To increase the recognition and prediction accuracy of machine learning, ensemble techniques are utilized (ML). By merging and pooling several subpar prediction models, these solutions often help to alleviate over-fitting concerns (component sub-models). These sub-models learn nonlinearly and intelligently, producing a learner that is more accurate at making predictions. The best parametric/predictive model is produced by combining qualifying sub-models using averaging or voting combination techniques.

#### Bagging and boosting algorithm

**Figure 9:**
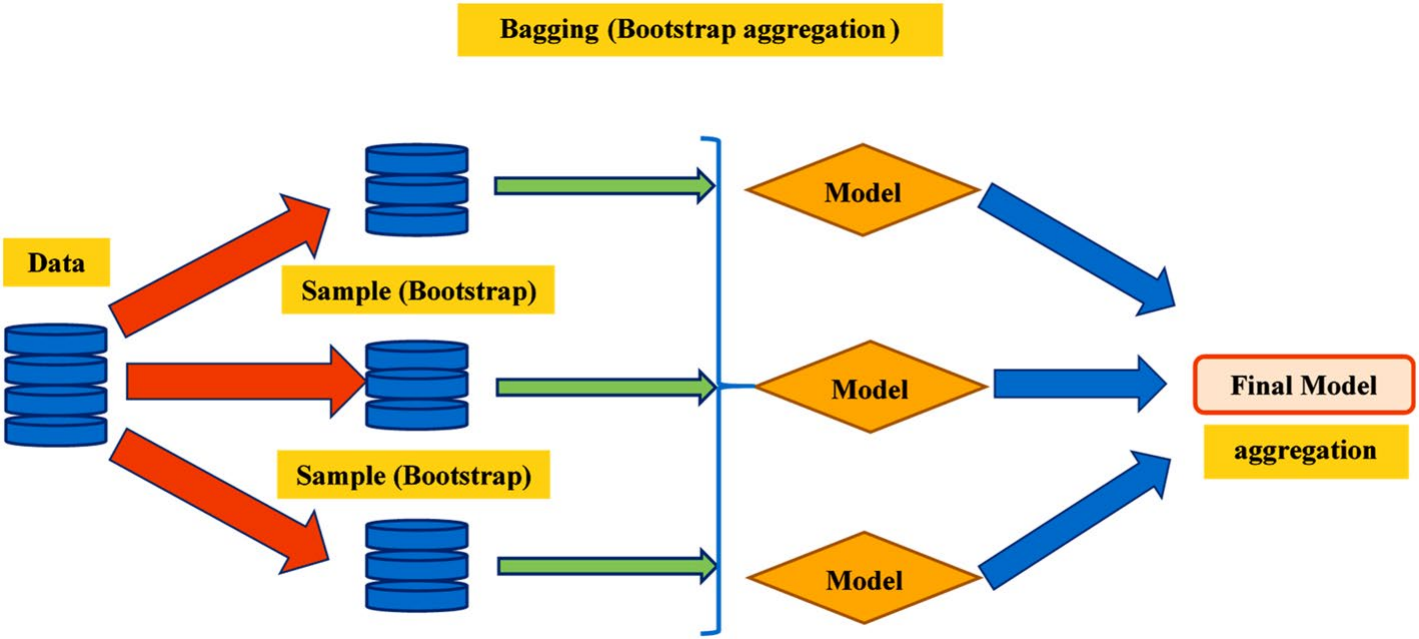
Bagging algorithm schematic chart.

Figure 9 depicts the schematic technique of the bagging model. Bagging is the term used to describe the modification to the estimation procedure resulting from the substitution of a new dataset for the training dataset. The primary set data are replaced as part of the strategy for irregular sampling. Using replacement sampling, specific observations can be replicated in each training database. Every phase of the bagging procedure must have an equal option in the new database. The training dataset size has no effect on the accuracy of predictions. Moreover, modifying the intended outcome prediction appropriately reduces variance significantly. Using this resource, additional models can be trained^84^. In this ensemble, the predicted values of the models are utilized. In regression, a prediction could be the mean of forecasts from multiple models. The optimal output value is determined by utilizing the DT's 20 sub-models to update the aggregating model. In addition the overall bagging principle of ensemble learning process involves the following steps. (1) Bootstrap Sampling: Random subsets of the original dataset are created through bootstrap sampling, where data points are randomly selected with replacement. Multiple subsets, often of equal size to the original dataset, are generated. Each subset is used to train a separate base model. (2) Model Training: A base model (e.g., decision tree, neural network) is trained independently on each subset of the data. Each model learns different patterns from its subset due to the randomness introduced by the sampling process. For instance, in the case of neural networks, multiple networks are trained with different subsets. (3) Individual Predictions: Once the models are trained, they are used to make predictions on the validation or test dataset (data not used for training). Each model independently generates its predictions based on the input data it has been trained on. (4) Aggregation of Predictions: The final output is generated by combining the predictions of all individual models. For regression tasks, this often involves averaging the predictions made by each model. In classification tasks, the final prediction may be determined by majority voting or averaging the probabilities predicted by each model. (5) Final Output: The aggregated predictions from all models form the final output of the bagging ensemble method. This combined output tends to be more robust and accurate compared to the prediction of any single model, as it leverages the diverse perspectives learned by each model from its subset of the data.

**Figure 10:**
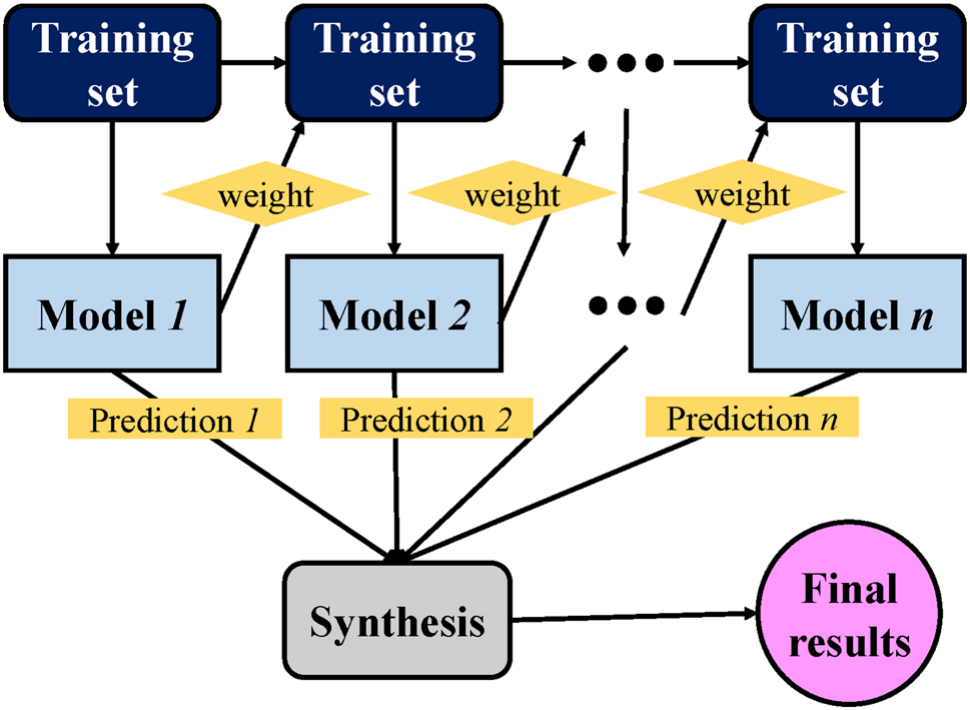
Boosting algorithm schematic chart.

Like the bagging technique, the boosting strategy influences the creation of several components that are more precise than a single model by creating a collective model as illustrated in Fig. 10. By means of biased averages of the dependent sub-models, the boosting approach chooses which sub-models to include in the final model. In this work, a boosting and bagging strategy was utilized to build an ensemble model on FASBGP concrete using a single ANN algorithm as a fundamental learner. Moreover, process regarding boosting as ensemble approaches involves the following steps. (1) Weighted Training: Initially, each data point in the training set is given equal weight. A weak learner (e.g., decision tree with limited depth) is trained on the entire dataset, focusing on the areas where the previous model(s) made mistakes. This model tries to fit the data while minimizing the errors. (2) Error Calculation: After the first model is trained, the errors or misclassifications made by this model are identified by comparing its predictions to the actual labels. Data points that were incorrectly predicted are assigned higher weights, while correctly predicted points are given lower weights. (3) Focus on Misclassified Data: The next weak learner is trained on the modified dataset, where the emphasis is placed on the previously misclassified data points. This iterative process aims to ‘boost’ the model’s performance by focusing on the areas where it has not performed well. (4) Sequential Improvement: The process continues for multiple iterations (typically with a predefined number of iterations or until a specified performance threshold is reached). Each subsequent model is trained to correct the errors of the combined ensemble of models created so far. (5) Weighted Combination: Finally, the predictions from all weak learners are combined with different weights assigned to each model based on its performance. Usually, models that perform better are given higher weights in the final prediction. (6) Final Output: The final output is generated by aggregating the predictions of all weak learners, often through a weighted sum or a weighted voting scheme, where the weights are determined by the performance of each model in the ensemble.

#### Parameter’s tuning for ensemble learner

The model tuning variables that are employed in making an ensemble approach can be (i) learning rate (ii) parameters associated with the finest number of model learners and other specific characteristics that have a significant influence on ensemble methods. The best and optimal model using twenty sub-model each with 10, 20, 30,…, 200, and nth estimator components on-base learner (ANN) were made and the most robust performance was selected. Table 5 demonstrates how the optimal model was selected using the correlation with high coefficient values. As a consequence, the ensemble with boosting outperforms the bagging and individual ANN models in terms of correlation coefficient.

**Table 5:** Sub-model details in making an ensemble model.

Approaches employed	Ensemble approach	Machine-learning methods	Ensemble sub models	Optimal estimator	R^2^-value
Individual	–	MLPNN	–	–	0.80
Ensemble	Bagging	Multilayer perceptron neuron network- Bagging	(10,20,30….200)	180	0.87
Ensemble	Boosting	Multilayer perceptron neuron network- Adaboost	(10,20,30….200)	170	0.89

### K-fold cross-validation method

**Figure 11:**
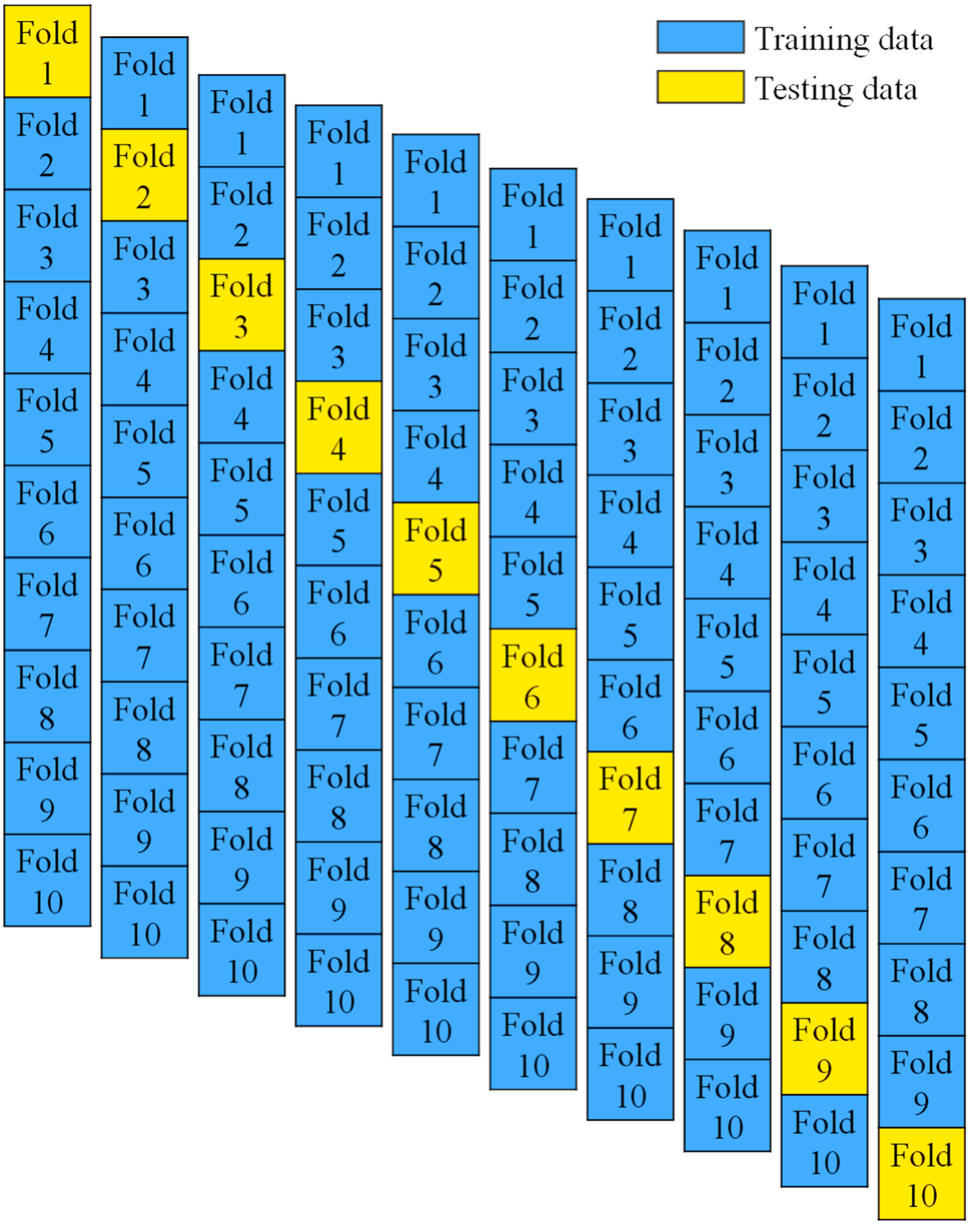
K-fold cross-validation.

Cross-validation (CV) is a technique that is used to examine and eliminate the bias and variance of the data to make an effective machine learning model. It is also known as a re-sampling procedure, and it is employed to assess the model’s effectiveness. This strategy is the easiest and outcomes in a less biased model as compared with other models. This is because it ensures that every observation from the data has an equal probability of appearing in both the test and train sets^85^. The accuracy of the algorithm is obtained in the form of statistical and average errors accuracy throughout ten verification cycles as demonstrated in Fig. 11.

### Statistical analysis

Statistical checks are employed to evaluate the efficacy and accuracy of the model. Mean absolute error (MAE), coefficient of determination (R^2^), root mean square error (RMSE), relative squared error (RSE), and relative root mean square error (RRMSE) are some statistical indicators. These indicators are defined by Eqs. (1–5).

1 $${R}^{2}= 1-\frac{\sum_{j=1}^{m}{({p}_{j}-{t}_{j})}^{2}}{\sum_{j=1}^{m}({t}_{j}-\overline{t })}$$

2 $$RMSE= \sqrt{\frac{\sum_{j=1}^{m}{\left({t}_{j}-{p}_{j}\right)}^{2}}{n}}$$

3 $$RRMSE= \frac{1}{\left|\overline{t }\right|}\sqrt{\frac{\sum_{j=1}^{m}{\left({t}_{j}-{p}_{j}\right)}^{2}}{n}}$$

4 $$MAE= \frac{\sum_{j=1}^{m}\left|{t}_{j}-{p}_{j}\right|}{n}$$

5 $$RSE=\frac{\sum_{j=1}^{m}{\left({p}_{j}-{t}_{j}\right)}^{2}}{\sum_{j=1}^{m}{\left(\overline{t }-{t}_{j}\right)}^{2}}$$

where ${t}_{j}$= Before creating a model, try out different values. ${p}_{j}$= The model's expected result. ${\overline{t} }_{j}$= Indicate the desired mean value. ${\overline{p} }_{i}$= show the anticipated mean value. $m$= Denotes the total number of examples used for modeling.

A higher coefficient of determination (R^2^) of the model is used to assess its reliability and accuracy, and a higher value with a lower value of statistical indicators indicates an ideal model^86^. R^2^ values are usually between 0 and 1, and a value close to 1 means that the experimental and predicted values are linked well^87^. According to some experts, the effectiveness of the proposed model significantly affects the model's ratio of data points to inputs. The optimal model should have a ratio larger than five to assess if the data points are acceptable for creating the required relationship between chosen variables. According to the present study's results, the proposed ratio is 17, which fits the researchers' standards. In addition, external statistical checks are used to assess the model, which are detailed in Table 6.

**Table 6:** Literature-suggested statistical metrics.

Equations	Condition	Recommended by
$k=\frac{{\sum }_{j=1}^{m}\left({t}_{j} \times {p}_{j}\right)}{{t}_{j}^{2}}$	0.85 < *k* < 1.15	^88^
$k{\prime}=\frac{{\sum }_{j=1}^{m}\left({t}_{j} \times {p}_{j}\right)}{{p}_{j}^{2}}$	0.85 < *k*′ < 1.15	^88^
${R}_{m}={R}^{2} \times \left(1-\sqrt{\left|{R}^{2}-{R}_{0}^{2}\right|}\right)$	*R*_*m*_ > 0.5	^89^
where		
${R}_{0}^{2}=1- \frac{\sum_{j=1}^{m}{\left({p}_{j}-{t}_{j}^{o}\right)}^{2}}{\sum_{j=1}^{m}{\left({p}_{j}-\overline{{p}_{j}^{o}}\right)}^{2} }; {t}_{j}^{o}=k\times {p}_{j}$	${R}_{o}^{2} \cong 1$	
${R}_{o}^{{\prime}2}=1- \frac{\sum_{j=1}^{m}{\left({t}_{j}-{p}_{j}^{o}\right)}^{2}}{\sum_{j=1}^{m}{\left({t}_{j}-\overline{{t}_{j}^{o}}\right)}^{2} }; {p}_{j}^{o}=k{\prime}\times {t}_{j}$	${R}_{o}^{{\prime}2} \cong 1$	

## Model result

**Figure 12:**
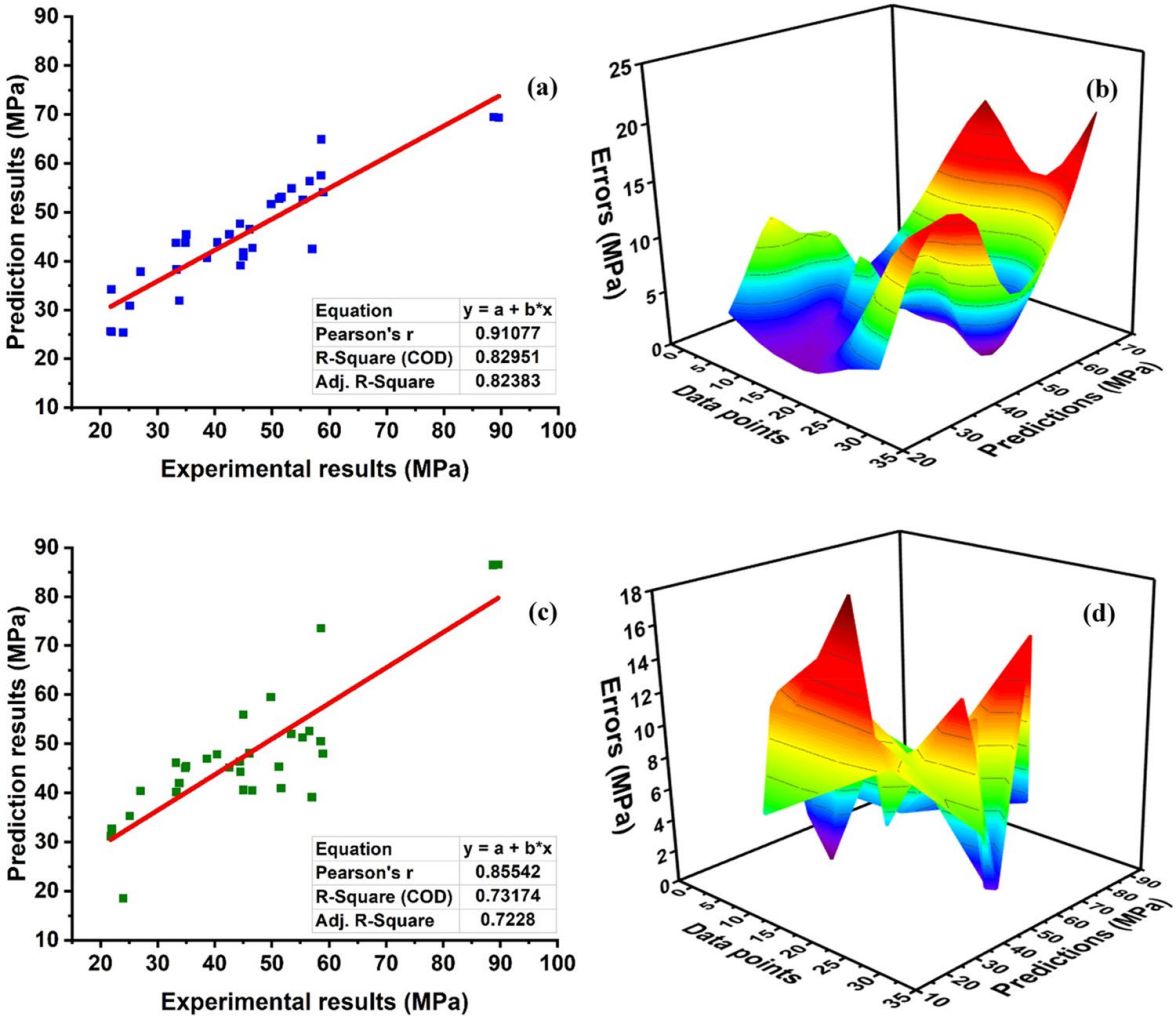
Regression models. (**a**) ANN prediction model; (**b**) error distribution of ANN model; (**c**) LR regression prediction model; (**d**) error distribution of LR model.

The prediction results from the supervised algorithms give robust performance as illustrated in Fig. 12. It can depicts in Fig. 12a that the ANN algorithm yield a good correlation with R^2^ = 0.82951 with better accuracy. This is because ANN uses hidden layers to give a better response as compared to linear regression. Moreover, the model accuracy of FASBC is also depicted by its errors allocation as in Fig. 12b. Figure 12a represents that some of the predicted data points lie away from the regression line and the same is also depicted in the error graph. In addition, a comparison of the model is made with linear regression (LR) in terms of R^2^ and error allocation as depicted in Fig. 12c,d. The evaluation of models is compared through MAE and the average errors of the testing set. ANN model shows 28 percent and 27% enhancement with MAE and average error respectively. This is due to the complex behavior of ANN as it mitigates the effect of linear regression and gives robustness performance.

### Ensemble with Adaboost algorithm

**Figure 13:**
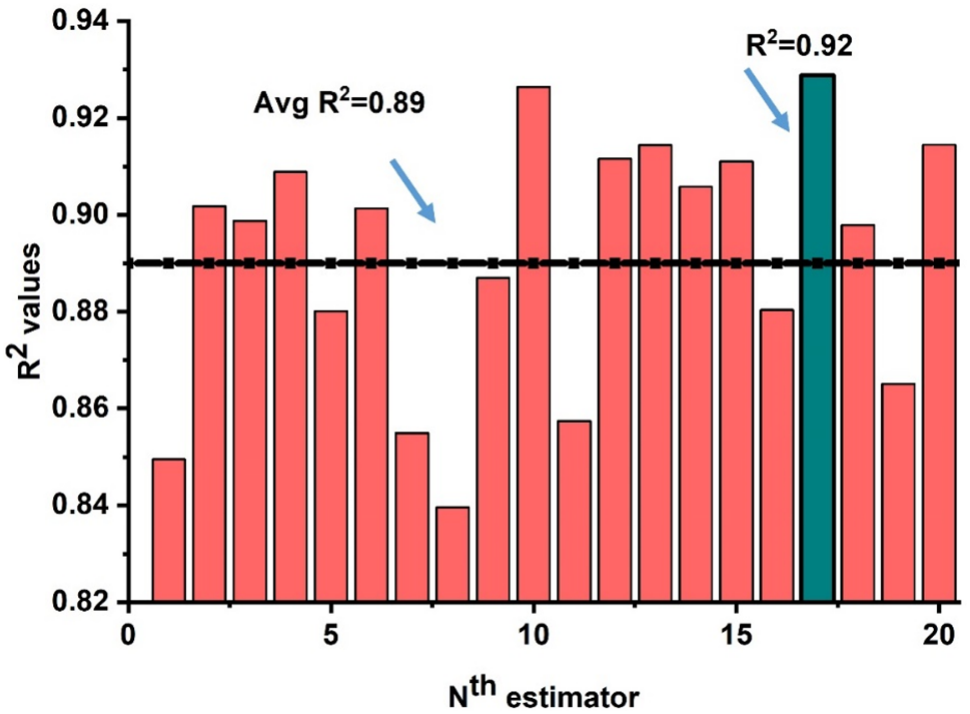
Regression models (Adaboost) prediction with twenty sub-models.

**Figure 14:**
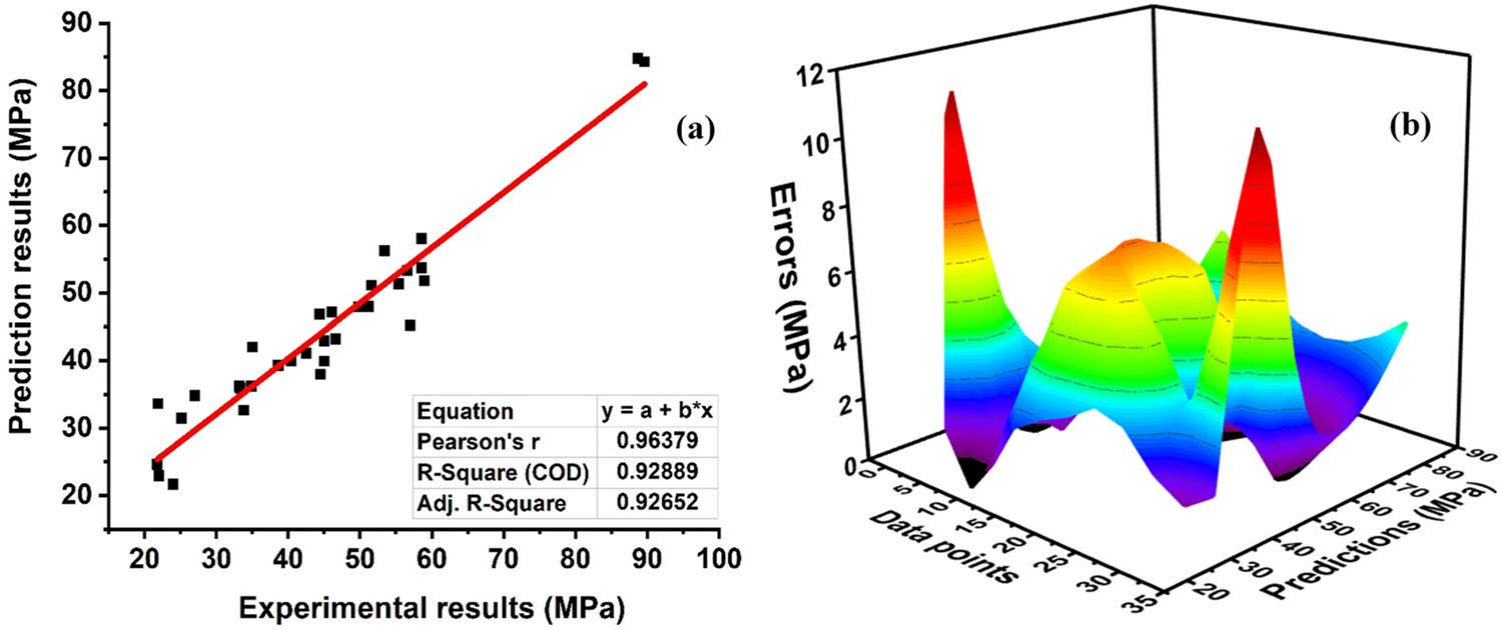
Regression models. (**a**) Adaboost prediction model; (**b**) error distribution of Adaboost model.

The prediction via ensemble algorithm using the Adaboost method depicts stronger correlations as compared to the individual model ANN and LR. This is due to the ensemble model as it takes into account the base ANN model to give the best optimal model as illustrated in Fig. 13. Twenty-sub models are constructed and an optimal model is selected as depicted in Fig. 14. It can be seen that Adaboost gives a robust performance of R^2^ = 0.92889 with seventeen sub-model as it takes several weak learners to predict the optimal response of the model (see Fig. 14a). Moreover, Furqan et al.^73^ anticipated the same response by employing ensemble algorithms. In addition, Fig. 14b demonstrates the error distribution of the ensemble model. It demonstrates that average errors are reduced and exhibits a 33 percent improvement over the individual ANN model. In addition, the Adaboost model illustrates 32 percent robust performance with MAE error than 51 percent for LR showing that experimental and predicted values are close to one with the least errors.

### Ensemble with bagging algorithm

**Figure 15:**
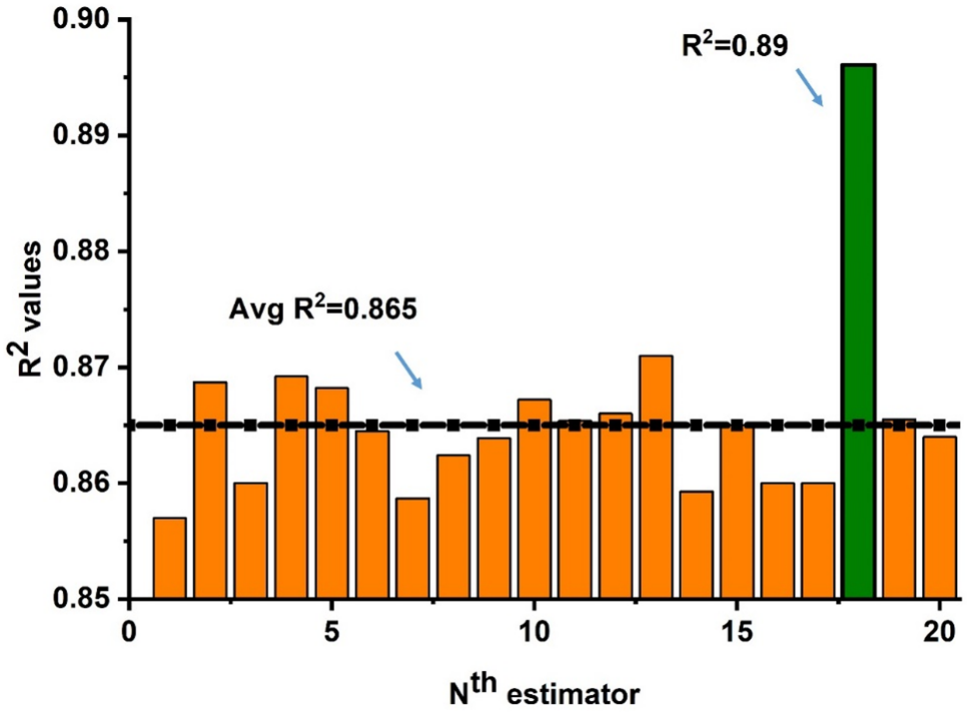
Regression models with (Adaboost) prediction with twenty sub-models.

**Figure 16:**
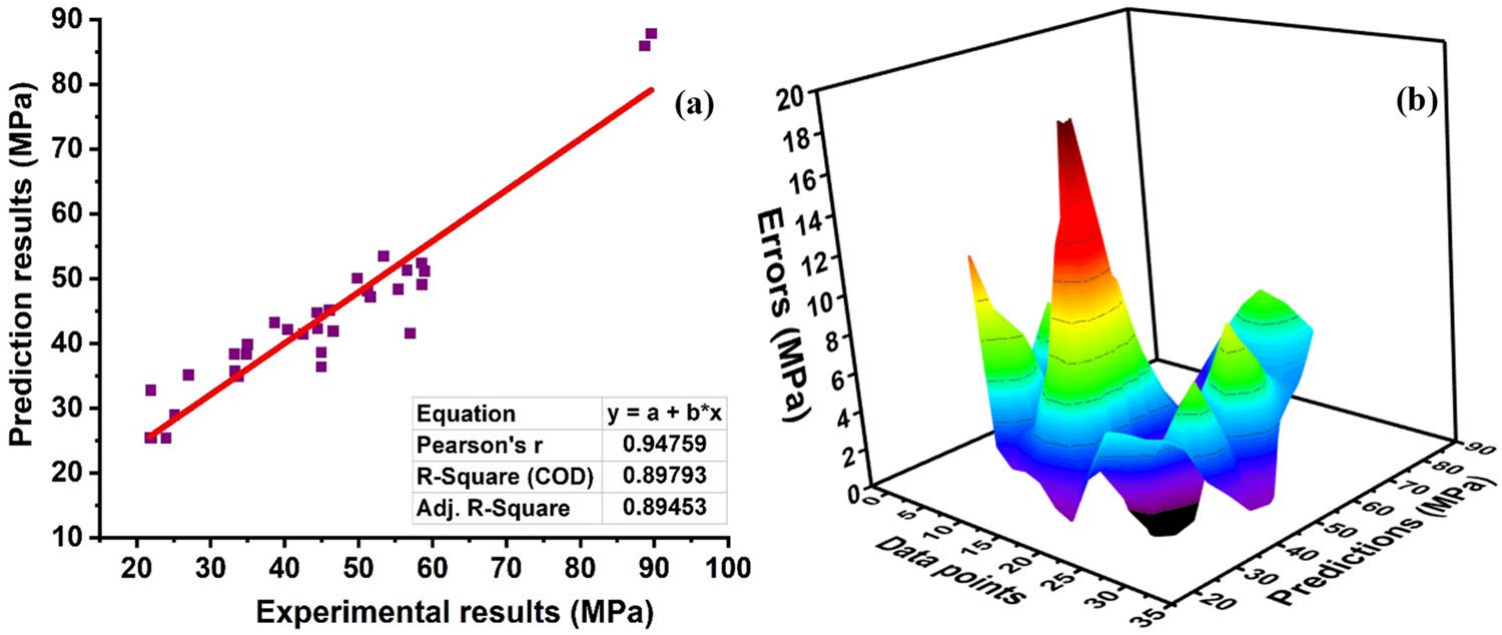
Regression models. (**a**) Bagging prediction model; (**b**) error distribution of bagging model.

The predicted outcome of FASBC with a bagging approach with twenty sub-models is illustrated in Fig. 15. It can be seen that the ensemble models show good performance from the individual ANN model as depicted in Fig. 16 with R^2^ = 0.89. Moreover, the bagging algorithm decreases the effect of variance and overfitting of data to give a better response. The individual model which is a weak learner is trained on twenty sub-models to give the most advantageous predicted outcome as depicted in Fig. 16. Figure 16a demonstrates that the submodels with n estimater equal to eighteen exhibit robust performance. In contrast, the model’s accuracy is also estimated by its mistakes, as illustrated in Fig. 16b. Furthermore, the bagging model outperforms ANN in terms of MAE and average error distribution, with a current accuracy of 51% when compared.

### Statistical analysis of model

Statistical analysis is used to assess the model’s effectiveness in predicting concrete properties. Moreover, it is the mathematical equation-based formalization of connections between variables in data. Furthermore, the coefficient of determination (R^2^), mean absolute error (MAE), relative squared error (RSE), root mean square error (RMSE), and relative root mean square (RRMSE) were used to compare individual and ensemble methods, as shown in Table 7.

**Table 7:** Statistical evaluation of models with different errors.

Approaches use	ML methods	Statistical measures	
Individual	Linear regression	R^2^	0.710
		RMSE	8.75
		RRMSE	0.20
		MAE	7.65
		RSE	0.29
Individual learner	ANN	R^2^	0.783
		RMSE	7.57
		RRMSE	0.17
		MAE	5.57
		RSE	0.22
Ensemble learning bagging	ANN-Bagging	R^2^	0.877
		RMSE	5.69
		RRMSE	0.12
		MAE	4.16
		RSE	0.12
Ensemble learning boosting	ANN-Adaboost	R^2^	0.914
		RMSE	4.75
		RRMSE	0.11
		MAE	3.74
		RSE	0.09

Moreover, an external statistical analysis check is also applied to the predicted outcome as mentioned in the literature. The result of the aforementioned algorithm is summarized in Table 8.

**Table 8:** Statistical evaluation of models using external checks.

Equations used to validate	Condition to obtain	Model used	Values
$k=\frac{{\sum }_{j=1}^{m}\left({t}_{j} \times {p}_{j}\right)}{{t}_{j}^{2}}$	0.85 < *k* < 1.15	ANN	0.946
		ANN-Bagging	0.981
		ANN-Adaboost	1.034
$k{\prime}=\frac{{\sum }_{j=1}^{m}\left({t}_{j} \times {p}_{j}\right)}{{p}_{j}^{2}}$	0.85 < *k*′ < 1.15	ANN	0.967
		ANN-Bagging	0.978
		ANN-Adaboost	1.008
${R}_{m}={R}^{2} \times \left(1-\sqrt{\left|{R}^{2}-{R}_{0}^{2}\right|}\right)$	*R*_*m*_ > 0.5	ANN	0.964
		ANN-Bagging	0.971
		ANN-Adaboost	0.120
${R}_{o}^{{\prime}2}=1- \frac{\sum_{j=1}^{m}{\left({t}_{j}-{p}_{j}^{o}\right)}^{2}}{\sum_{j=1}^{m}{\left({t}_{j}-\overline{{t}_{j}^{o}}\right)}^{2} }; {p}_{j}^{o}=k{\prime}\times {t}_{j}$	${R}_{o}^{{\prime}2} \cong 1$	ANN	0.997
		ANN-Bagging	0.999
		ANN-Adaboost	0.999

### Cross-validation of K-folds

**Figure 17:**
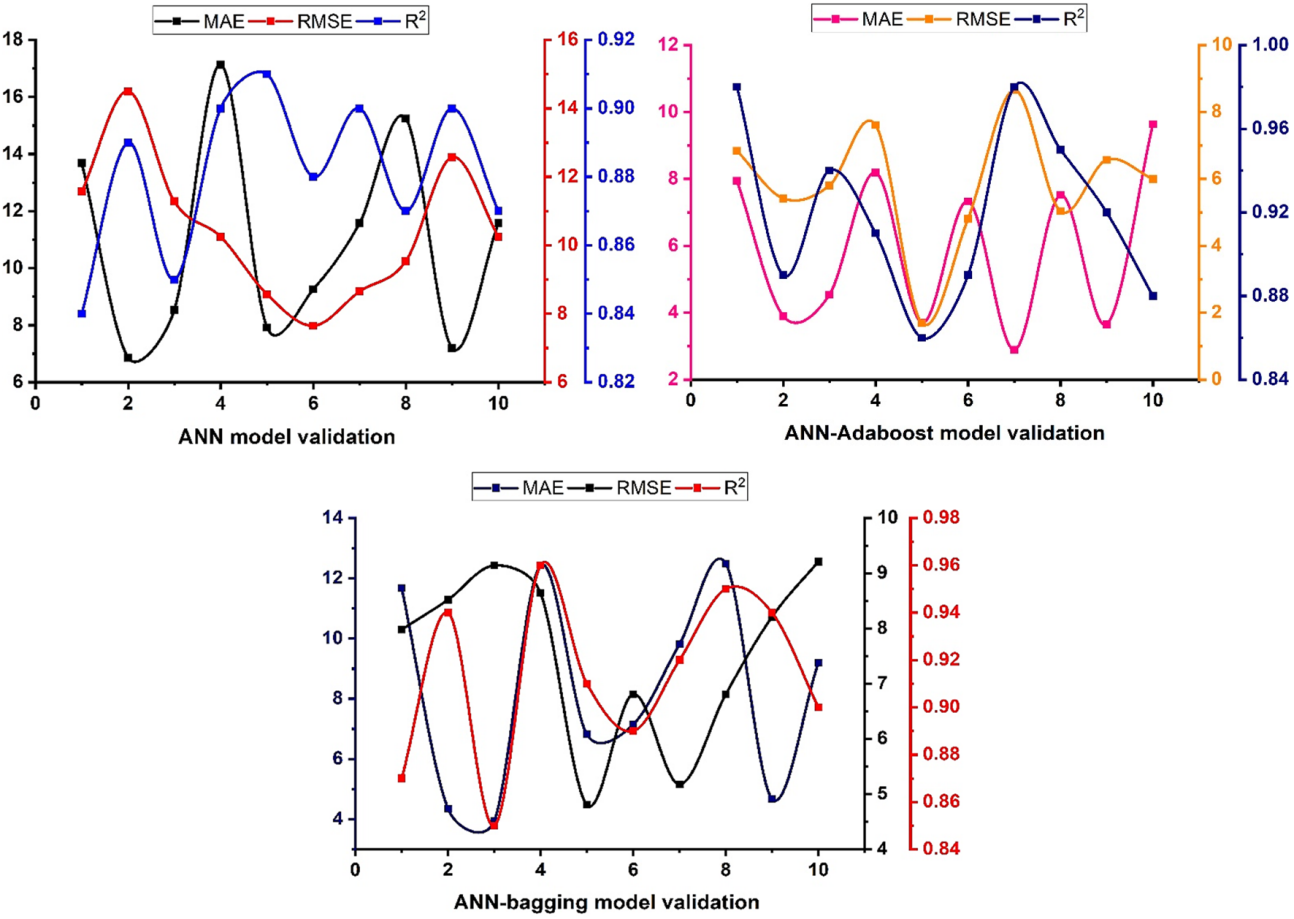
K-fold validation approach.

The validation process is essential to the model's success because it ensures that the data used to create the model is as accurate as possible. K-Fold CV is employed to improve the reliability, robustness, and effectiveness of the individual and ensemble models as depicted in Table 9 and Fig. 17. It can be seen that the ANN model with Adaboost outclassed with individual ANN and ANN with bagging model. Moreover, a comparison is made between its average error using MAE and RMSE. MAE of ANN-AdaBoost gives enhanced results by about 46% and ANN-bagging depicts 24% robust performance. Similarly, boosting and bagging show a 44% and 28% increase in model capacity as compared to the individual ANN model. The same result has been reported by Ayaz et al.^70^ by employing ensemble approaches to predict the most beneficial outcome.

**Table 9:** Evaluation of models using K-Fold validation.

K-fold	ANN			ANN-Adaboost			ANN-bagging		
	MAE	RMSE	R^2^	MAE	RMSE	R^2^	MAE	RMSE	R^2^
1	13.68	11.57	0.84	7.94	6.8386	0.98	11.68	7.98	0.87
2	6.86	14.5	0.89	3.9	5.41552	0.89	4.35	8.52	0.94
3	8.54	11.28	0.85	4.54119	5.80112	0.94	3.94	9.14	0.85
4	17.14	10.25	0.9	8.18209	7.60701	0.91	12.42	8.64	0.96
5	7.92	8.57	0.91	3.70382	1.689	0.86	6.82	4.81	0.91
6	9.258	7.65	0.88	7.32827	4.812	0.89	7.14	6.81	0.89
7	11.578	8.65	0.9	2.89234	8.6571	0.98	9.81	5.18	0.92
8	15.24	9.54	0.87	7.518	5.034	0.95	12.48	6.81	0.95
9	7.2	12.57	0.9	3.654	6.571	0.92	4.68	8.21	0.94
10	11.58	10.25	0.87	9.63297	5.99667	0.88	9.2	9.21	0.9

## Shapley analysis

**Figure 18:**
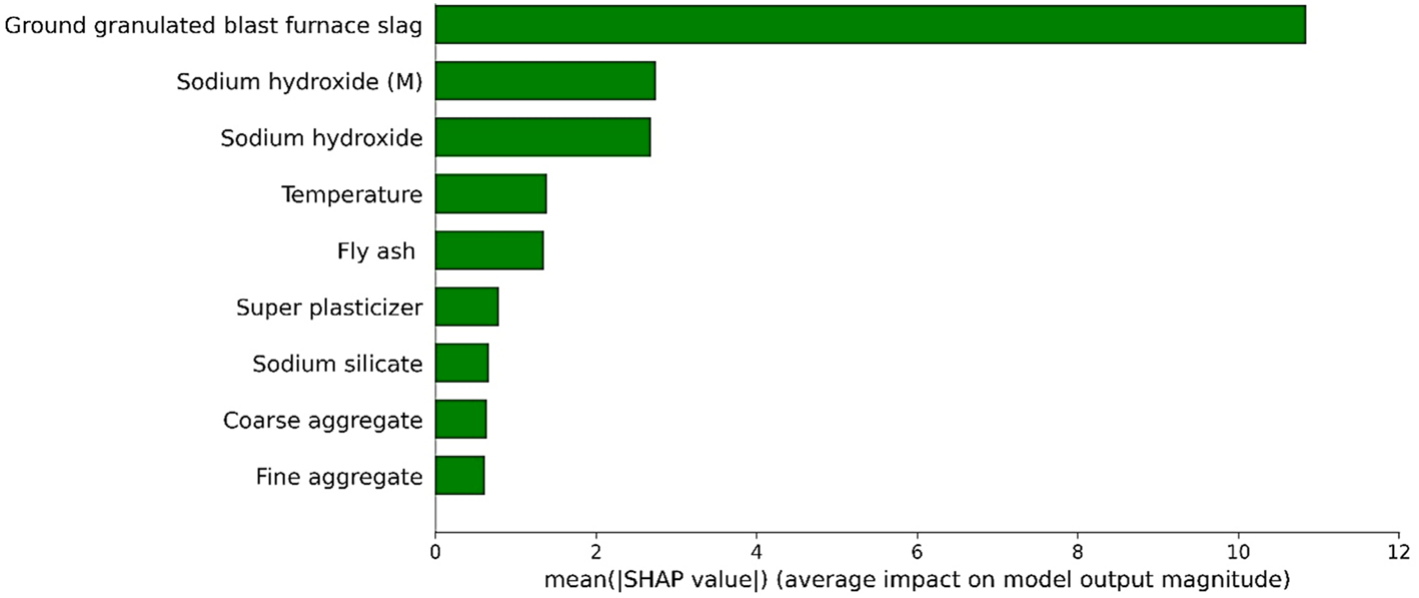
Shapley analysis of FASBC.

**Figure 19:**
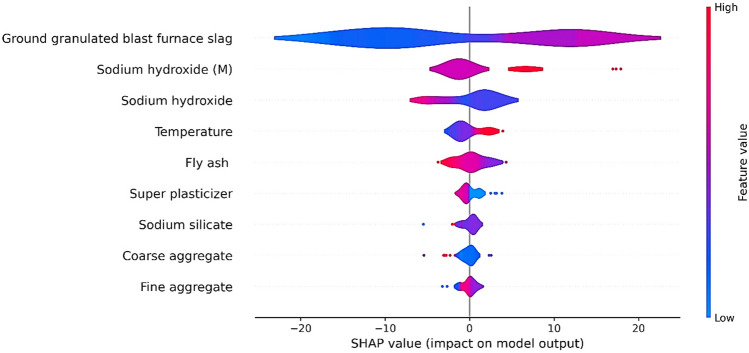
Feature impact on model.

The shapley analysis is performed on FASBC using the Sklearn library in Python and the detailed analysis of each variable is illustrated in Fig. 18. In addition, the best method is to firstly apply the SHAP explainer to the training data subgroup to recognize the model's behavior and address any issues, followed by validation on the testing data subset to evaluate interpretability and generalization performance. This comprehensive approach ensures a thorough evaluation of the model's behavior across different datasets and leads to more robust interpretations. It demonstrates that GGBS, temperature, sodium hydroxide and its molarity, and FA have a significant impact on the compressive strength of FASBC. This is due to the presence of Al_2_O_3_, CaO, and SiO_2_ in the amorphous state^90–92^. Furthermore, GGBS has a binding behavior in an alkaline media. It's a key ingredient in geopolymer concrete as it contributes to its strength and durability. GGBS contains silicates and aluminates, which react with the alkali activator (sodium hydroxide) to form the binding matrix in geopolymer concrete^93^. Similarly, temperature affects the curing process of geopolymer concrete. Generally, higher curing temperatures can accelerate the reaction between the alkaline solution (usually sodium hydroxide) and the precursors (like GGBS and fly ash), leading to faster setting times and increased strength. Moreover, sodium hydroxide, commonly known as caustic soda, is used as an alkaline activator in geopolymer concrete. Its concentration (molarity) influences the reactivity and strength of the resulting geopolymer. Higher concentrations or molarities of sodium hydroxide often lead to faster reaction rates and higher strengths^94^. Likewise, when GGBS is mixed with FA in an alkaline media, extra calcium content is formed, which is responsible for the improved mechanical qualities. Thus, contributes to the overall strength and durability of the concrete. Local and global explainability of the model was enhanced using SHAP analysis. In global explainability analysis, mean SHAP values were used for features importance ranking as depicted in Fig. 18, and a summary plot was used to indicate the features values’ impact on the CS as shown in Fig. 19. The summary plots display the trend of the associated variable and the distribution of SHAP values for a certain feature. The y-axis of the summary plot displays the input variables utilized in the study and their significance in a sequential manner, while the x-axis represents the corresponding SHAP value. The dark color represents their size, ranging from little (blue) to large (red), and they serve as samples in the database. The x-axis represents the range of prediction, measured by SHAP values, for each variable as the input variables change in magnitude (from blue to red). In addition, red indicates positive SHAP values, suggesting that higher values of that feature positively impact the model's output or prediction. For instance, higher values might lead to higher predicted outcomes. Simiarly, blue represents negative SHAP values, implying that lower values of that feature have a positive effect on the model's output. In other words, lower values might result in higher predicted outcomes. It can be seen that all the feauture have impact on model performance with GGBS have major influence. The strength increases proportionally with the ground granulated blast furnace slag (GGBS) content in the specimen. FA exhibits red color values on the left side and blue color values on the right-hand side. These findings indicate that elevating FA above a specific threshold can result in a reduction in CS. Furthermore, elevating the temperature and utilizing a greater concentration of Na_2_SiO_3_ can positively influence the compressive strength (CS) of GPC. Nevertheless, the amount of sodium hydroxide (NaOH) had both beneficial and detrimental effects on the CS, indicating the need to employ NaOH within the ideal range.

**Figure 20:**
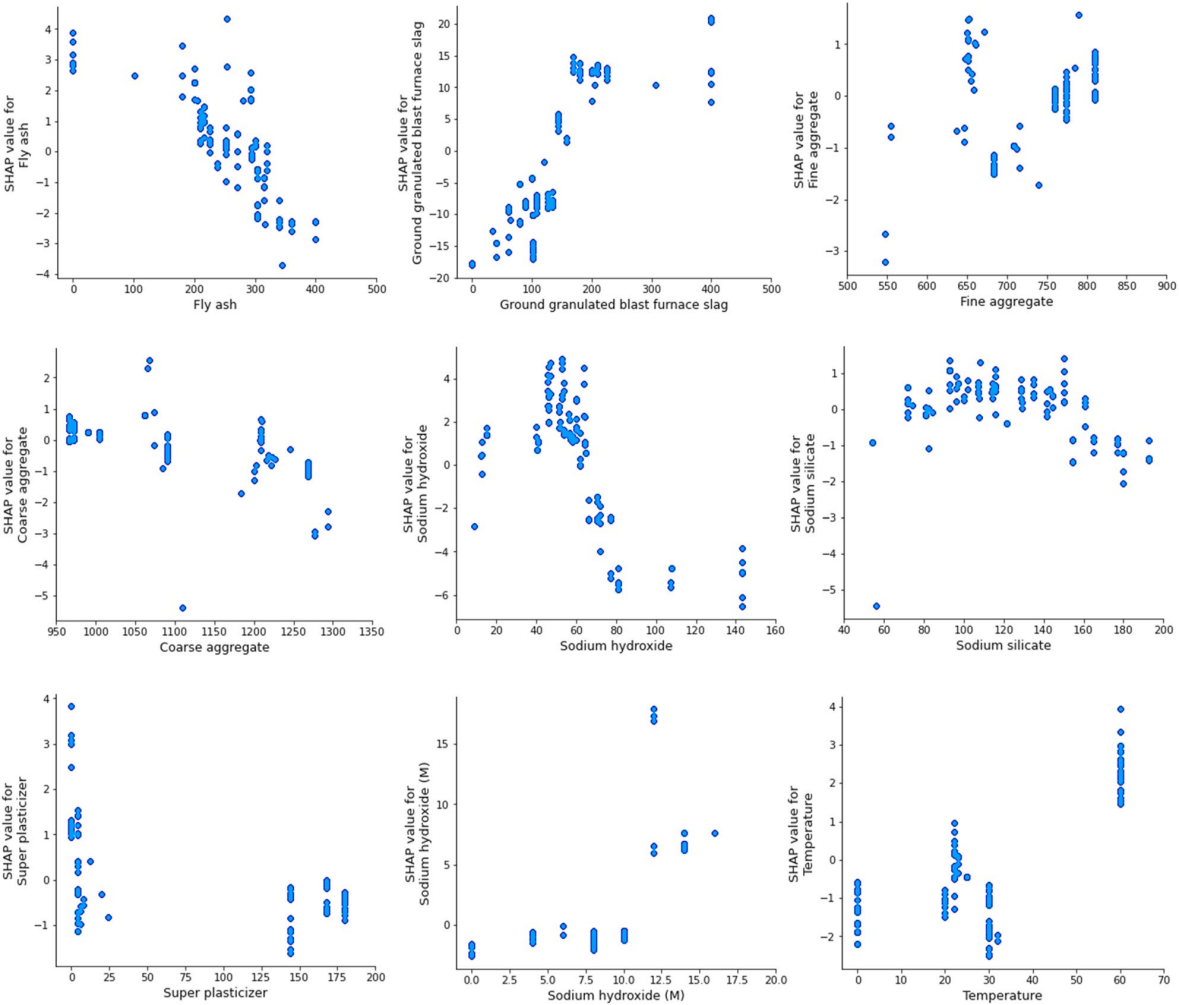
Features interaction with strength.

Figure 20 explores the correlation between components and their influence on GPC model. The graph demonstrates that increasing the FA results in a decrease in the CS after surpassing a threshold of 300 (kg/m^3^), indicating a substantial reduction in the CS. The SHAP study revealed that the ideal amount of FA was approximately 300 kg/m^3^, leading to the highest level of CS. However, the negative impact was demonstrated with higher levels of FA concentration. The negative effect on the majority of fly ash (FA) could be attributed to the disparity in calcium levels seen in the FA used across several investigations^95^. Furthermore, there is a notable upward tendency for GGBS, where higher concentrations greatly improve the compressive strength (CS) of GPC. Furthermore, Kashifi et al.^96^ has also seen a comparable pattern. Furthermore, both sodium silicate and sodium hydroxide exhibit a favorable impact up to a specific threshold. For example, when sodium silicate is elevated over a particular threshold, it has a harmful impact. Likewise, the effect of large-sized aggregate has a fluctuating impact on compressive strength, particularly when its quantity exceeds 1200 kg/m^3^. The data demonstrates that variables such as temperature and sodium hydroxide have a positive impact on the outcome of the model. It accelerates the polymerization reaction. Increasing the curing temperature was found to positively impact the compressive strength (CS) of polymer composites. Similary, Zhang et al.^97^ also documented a comparable effect. The findings of this study were obtained by analyzing the inputs and dataset size used in the SHAP analysis. Increasing the scope of the database to include a wider variety of input variables has the potential to yield more precise correlations.

## Graphical user interface

**Figure 21:**
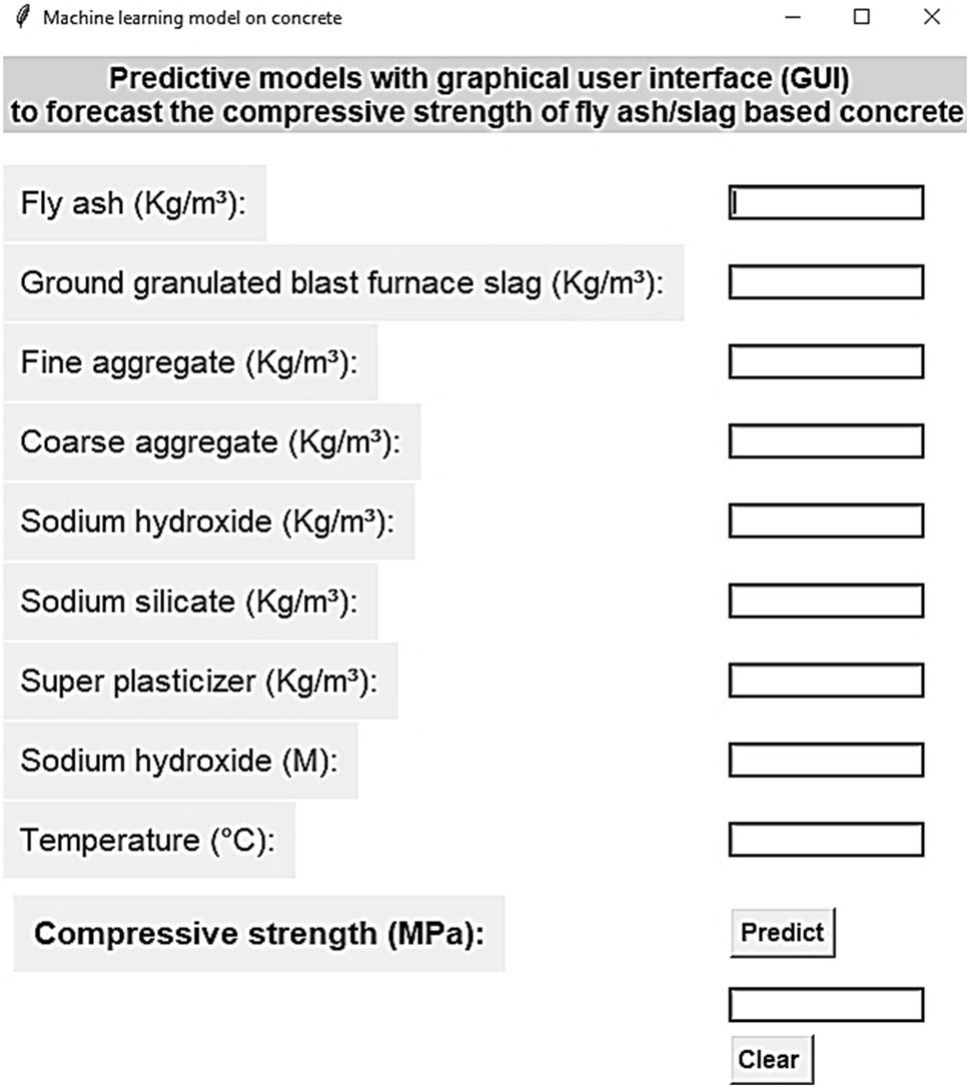
Graphical user interface of FASBC.

A straightforward and user-friendly graphical user interface with input variables and output strength has been built for the practical application of our model, as illustrated in Fig. 21. The user must input the amounts of the input variables in the units specified while the compressive strength will be calculated at the conclusion of the analysis. All settings may be reset to default empty values using the clear button. This straightforward GUI offers effective model applicability for both study and business. The creation of a graphical user interface (GUI), which offers a natural and user-friendly manner to interact with computer programs via visual components, is essential for the development of contemporary computer applications.

## Conclusion

The implementation of individual and ensemble methods employing ANN with Adaboost and bagging to anticipate the mechanical characteristics of FASBGPC is described in this study. Python code was utilized in the Anaconda prompt navigator with Spyder to run the necessary models for additional investigation. For the assessment and correctness of each model, statistical indicator checks in the form of numerous statistical mistakes were performed. The research led to the following findings:

1. The ensemble methods, which include bagging and boosting techniques, exhibited more dependable performance in comparison to individual methods. More precisely, the utilization of boosting with artificial neural networks (ANN) led to an enhancement compared to the individual strategy. Furthermore, both techniques demonstrated a strong association, with an R^2^ value exceeding 0.85.
2. Compared to the LR model, the ANN model's prediction of FASBGPC's compressive strength has a good relationship with R^2^ = 0.829. In addition, statistical indicator in form of MAE depicts that ANN models give 28% more accurate result. This shows that non-linear regression gives a robust performance than linear models.
3. Adaboost with ANN as an ensemble model shows an obstinate correlation of R^2^ = 0.928 as compared to ANN. Similarly, ANN with bagging gives R^2^ = 0.89, as opposed to ANN which is R^2^ = 0.829. Thus, the ANN model with boosting shows the least error and accurate performance than other models.
4. Numerical errors in the form of MAE, RMSE, RRMSE, RSE, and R^2^ provide obstinate outcomes for ANN with Adaboost by displaying the least error indications. Also, External validation checks fulfill the criteria and thus boosting present a good model.
5. Validation by using K-Fold cross-validation and statistical indicator shows a higher accuracy of the models.
6. Shapley analysis reveals that GGBS, sodium hydroxide molarity, and temperature has a major influential aspect on crushing strength of GPC.

### Supplementary Information

Supplementary Information.

## Data Availability

The datasets used during the current study available from the corresponding author on reasonable request.

## Code availability

The python-based source code that support the findings of this study is available at https://github.com/Kiranarif001/Paper-data.
