# Supplementary material for: Estimation of compressive strength of waste concrete utilizing fly ash/slag in concrete with interpretable approaches: optimization and graphical user interface (GUI)
Source: Sci Rep. 2024 Feb 26;14:4598. doi: 10.1038/s41598-024-54513-y (PMC10897462; doi:10.1038/s41598-024-54513-y)
Supplement: Supplementary file 1 — Supplementary Information. [file 41598_2024_54513_MOESM1_ESM.docx]

1. **MLA execution in anaconda python**

**Figure 1** depicts the model execution in graphical form. This will clarify our approach and make it simple for experts and researchers to accept it. Therefore, machine learning will perform well when used in the civil sector.

**Figure 1: Model execution process**

1. **Importing libraries to create a model**

The models were developed using the software tools Spyder and Jupyter Notebook, which include a number of packages including import numpy as np, import pandas as pd, seaborn, and scikit-learn libraries. Information about each library may be found in **Table 1**. There are two ways to set up the three regression models. The first technique is to type "pip install regression name" at the Anaconda prompt. The models are also available for download from the "Github" repository.

1. **Importing CSV data into the Anaconda database**

The published literature data was utllized for modlling of CS nanocomposite.this datset includes 205 data points with different concentration as shown in Annexure A. In addition, loading of libraries into Anaconda Python can be seen in **Table 1**. The "waste incorporated in concrete" data is then split into variables that are input and variables that are output. The "waste incorporated in concrete" data is then split into variables that are input and variables that are output.

1. **Data separation into the train and test sets**

A training set and a testing set are two subsets of the whole data collection. The characteristics and hyper-parameters that will lower prediction error are found using the training data. The best features and hyper-parameters are used to build the final model. The results of testing data are then predicted using it. For MLA modeling, the testing and training dataset set consists of 41 and 164 randomly chosen samples.The performance of the model is then evaluated using 10k fold cross validation.

1. **Performance evaluation**

This section examines statistical metrics that aid in determining a model's performance. Less mistakes and a higher R^2^ can indicate exceptional excellent performance.

**Table 1: Libraries detail**

**Figure 2** represents the scientometric review of the importance of machine learning algorithms (MLA) in cementitious composite


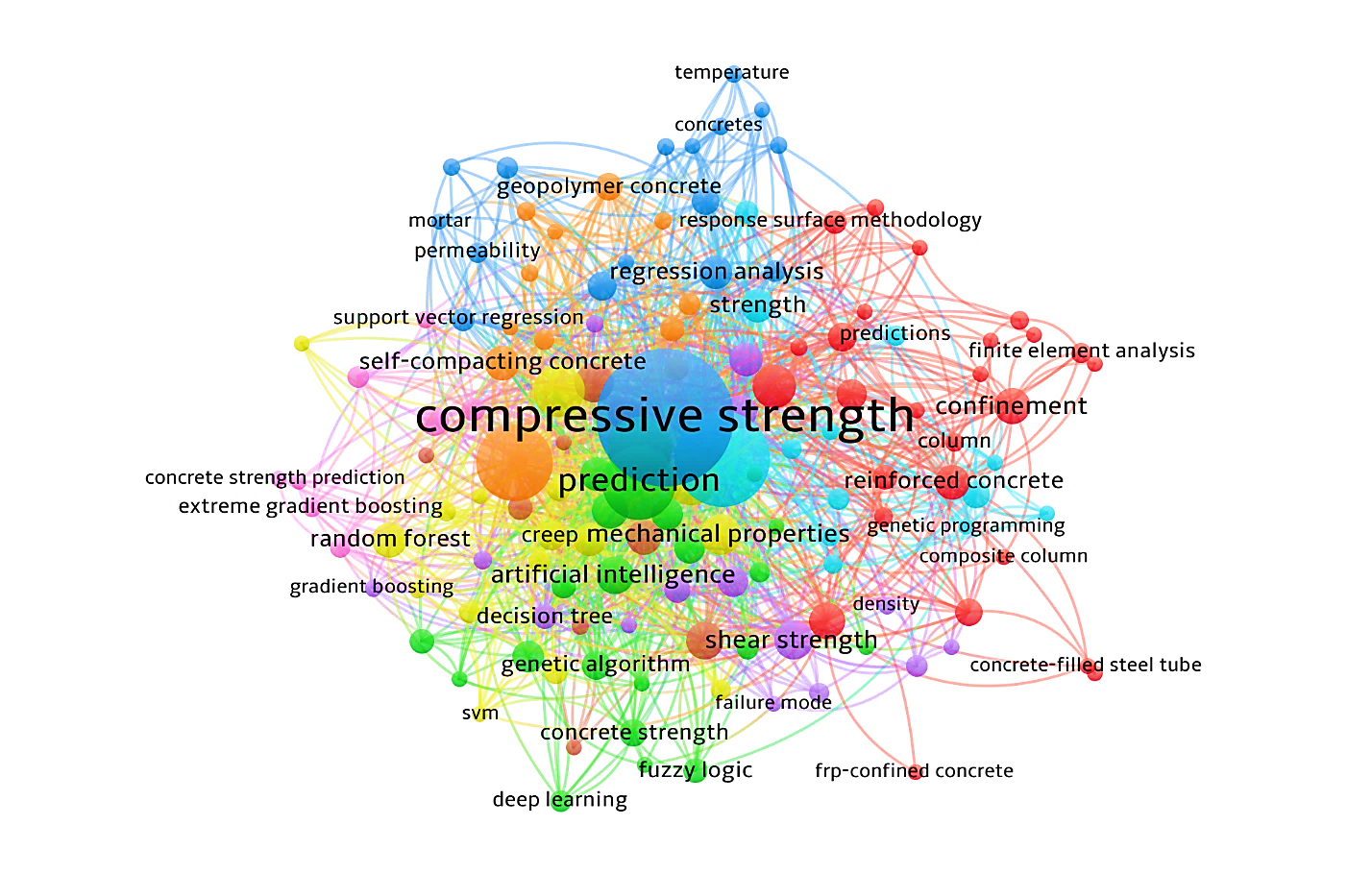


**Figure 2: MLA Scientometrics review in concrete**

**Figure 3: Boosting 20 models**

**Figure 4: Bagging 20 models**

**Figure 5: K-Fold cross validation individual results**

**Parameter Optimization Steps for Artificial Neural Network (ANN):**

**Hidden Layer**: A range of hidden layer sizes (from 2 to 40) was explored to assess the model's sensitivity to different network architectures.

**Max Iterations**: The maximum number of iterations was set to 450 to ensure convergence and prevent premature termination during training.

**Solver**: Multiple solvers (sgd, lbfgs, adam) were considered, with 'adam' identified as the optimal choice for optimizing weights.

**Activation Function**: Various activation functions (identity, logistic, tanh, relu) were evaluated, and 'relu' was determined to be the most suitable for our model.

**Learning Rate Initialization**: Different initial learning rates (ranging from 0.01 to 0.2) were tested, with a learning rate of 0.1 yielding optimal performance.

**Learning Rate Schedule**: Considering various learning rate schedules (constant, invscaling, adaptive), the 'constant' schedule was selected for stability.

**Ensemble Methods (Bagging and Boosting)**:

Similar hyperparameter tuning was conducted for bagging and boosting applied to the ensemble models. Parameters such as the number of estimators, learning rates (where applicable), and other relevant parameters were adjusted through a systematic search.

**Optimal Parameter Selection**:

The optimal values presented in the table and Figure represent the parameters that produced the best-performing model in terms of the chosen evaluation metric i.e (R^2^) after rigorous experimentation and validation. For Bagging and Boosting see **Figure 8**, **Figure 13**, **Figure 15** with N^th^ estimator with R^2^

**Data set**

| S.No | FA | GGBS | Fine | Coarse | NaOH | Na2SiO3 | SP | NaOH | Temp | Compressive strength |
| --- | --- | --- | --- | --- | --- | --- | --- | --- | --- | --- |
| 1 | 252 | 108 | 774 | 1090.8 | 46.3 | 115.7 | 144 | 8 | 30 | 33.83 |
| 2 | 252 | 108 | 774 | 1090.8 | 51.4 | 128.6 | 144 | 8 | 30 | 36.19 |
| 3 | 252 | 108 | 774 | 1090.8 | 56.6 | 141.4 | 144 | 8 | 30 | 31.11 |
| 4 | 252 | 108 | 774 | 1090.8 | 61.7 | 154.3 | 144 | 8 | 30 | 25.71 |
| 5 | 294 | 126 | 810.6 | 966 | 54 | 135 | 168 | 8 | 30 | 36.69 |
| 6 | 294 | 126 | 810.6 | 966 | 60 | 150 | 168 | 8 | 30 | 38.16 |
| 7 | 294 | 126 | 810.6 | 966 | 66 | 165 | 168 | 8 | 30 | 26.17 |
| 8 | 294 | 126 | 810.6 | 966 | 72 | 180 | 168 | 8 | 30 | 25.11 |
| 9 | 315 | 135 | 760.5 | 972 | 57.9 | 144.6 | 180 | 8 | 30 | 33.81 |
| 10 | 315 | 135 | 760.5 | 972 | 64.3 | 160.7 | 180 | 8 | 30 | 38.96 |
| 11 | 315 | 135 | 760.5 | 972 | 70.7 | 176.8 | 180 | 8 | 30 | 25.71 |
| 12 | 315 | 135 | 760.5 | 972 | 77.1 | 192.9 | 180 | 8 | 30 | 20.76 |
| 13 | 216 | 144 | 774 | 1090.8 | 46.3 | 115.7 | 144 | 8 | 30 | 42.32 |
| 14 | 216 | 144 | 774 | 1090.8 | 51.4 | 128.6 | 144 | 8 | 30 | 47.92 |
| 15 | 216 | 144 | 774 | 1090.8 | 56.6 | 141.4 | 144 | 8 | 30 | 46.87 |
| 16 | 216 | 144 | 774 | 1090.8 | 61.7 | 154.3 | 144 | 8 | 30 | 43.38 |
| 17 | 252 | 168 | 810.6 | 966 | 54 | 135 | 168 | 8 | 30 | 46.08 |
| 18 | 252 | 168 | 810.6 | 966 | 60 | 150 | 168 | 8 | 30 | 51.22 |
| 19 | 252 | 168 | 810.6 | 966 | 66 | 165 | 168 | 8 | 30 | 44.67 |
| 20 | 252 | 168 | 810.6 | 966 | 72 | 180 | 168 | 8 | 30 | 46.57 |
| 21 | 270 | 180 | 760.5 | 972 | 57.9 | 144.6 | 180 | 8 | 30 | 45.88 |
| 22 | 270 | 180 | 760.5 | 972 | 64.3 | 160.7 | 180 | 8 | 30 | 50.69 |
| 23 | 270 | 180 | 760.5 | 972 | 70.7 | 176.8 | 180 | 8 | 30 | 41.85 |
| 24 | 270 | 180 | 760.5 | 972 | 77.1 | 192.9 | 180 | 8 | 30 | 45.68 |
| 25 | 180 | 180 | 774 | 1090.8 | 46.3 | 115.7 | 144 | 8 | 30 | 55.37 |
| 26 | 180 | 180 | 774 | 1090.8 | 51.4 | 128.6 | 144 | 8 | 30 | 59.79 |
| 27 | 180 | 180 | 774 | 1090.8 | 56.6 | 141.4 | 144 | 8 | 30 | 51.61 |
| 28 | 180 | 180 | 774 | 1090.8 | 61.7 | 154.3 | 144 | 8 | 30 | 46.68 |
| 29 | 210 | 210 | 810.6 | 966 | 54 | 135 | 168 | 8 | 30 | 56.86 |
| 30 | 210 | 210 | 810.6 | 966 | 60 | 150 | 168 | 8 | 30 | 60.38 |
| 31 | 210 | 210 | 810.6 | 966 | 66 | 165 | 168 | 8 | 30 | 46.48 |
| 32 | 210 | 210 | 810.6 | 966 | 72 | 180 | 168 | 8 | 30 | 47.57 |
| 33 | 225 | 225 | 760.5 | 972 | 57.9 | 144.6 | 180 | 8 | 30 | 48.91 |
| 34 | 225 | 225 | 760.5 | 972 | 64.3 | 160.7 | 180 | 8 | 30 | 58.53 |
| 35 | 225 | 225 | 760.5 | 972 | 70.7 | 176.8 | 180 | 8 | 30 | 48.45 |
| 36 | 225 | 225 | 760.5 | 972 | 77.1 | 192.9 | 180 | 8 | 30 | 47.64 |
| 37 | 252 | 108 | 774 | 1090.8 | 46.3 | 115.7 | 144 | 8 | 60 | 41.53 |
| 38 | 252 | 108 | 774 | 1090.8 | 51.4 | 128.6 | 144 | 8 | 60 | 42.56 |
| 39 | 252 | 108 | 774 | 1090.8 | 56.6 | 141.4 | 144 | 8 | 60 | 33.94 |
| 40 | 252 | 108 | 774 | 1090.8 | 61.7 | 154.3 | 144 | 8 | 60 | 28.58 |
| 41 | 294 | 126 | 810.6 | 966 | 54 | 135 | 168 | 8 | 60 | 40.41 |
| 42 | 294 | 126 | 810.6 | 966 | 60 | 150 | 168 | 8 | 60 | 43.5 |
| 43 | 294 | 126 | 810.6 | 966 | 66 | 165 | 168 | 8 | 60 | 31.24 |
| 44 | 294 | 126 | 810.6 | 966 | 72 | 180 | 168 | 8 | 60 | 26.89 |
| 45 | 315 | 135 | 760.5 | 972 | 57.9 | 144.6 | 180 | 8 | 60 | 38.63 |
| 46 | 315 | 135 | 760.5 | 972 | 64.3 | 160.7 | 180 | 8 | 60 | 44.49 |
| 47 | 315 | 135 | 760.5 | 972 | 70.7 | 176.8 | 180 | 8 | 60 | 35.2 |
| 48 | 315 | 135 | 760.5 | 972 | 77.1 | 192.9 | 180 | 8 | 60 | 29.46 |
| 49 | 216 | 144 | 774 | 1090.8 | 46.3 | 115.7 | 144 | 8 | 60 | 55.57 |
| 50 | 216 | 144 | 774 | 1090.8 | 51.4 | 128.6 | 144 | 8 | 60 | 57.37 |
| 51 | 216 | 144 | 774 | 1090.8 | 56.6 | 141.4 | 144 | 8 | 60 | 53.39 |
| 52 | 216 | 144 | 774 | 1090.8 | 61.7 | 154.3 | 144 | 8 | 60 | 51.22 |
| 53 | 252 | 168 | 810.6 | 966 | 54 | 135 | 168 | 8 | 60 | 58.8 |
| 54 | 252 | 168 | 810.6 | 966 | 60 | 150 | 168 | 8 | 60 | 62.29 |
| 55 | 252 | 168 | 810.6 | 966 | 66 | 165 | 168 | 8 | 60 | 58.93 |
| 56 | 252 | 168 | 810.6 | 966 | 72 | 180 | 168 | 8 | 60 | 56.64 |
| 57 | 270 | 180 | 760.5 | 972 | 57.9 | 144.6 | 180 | 8 | 60 | 56.56 |
| 58 | 270 | 180 | 760.5 | 972 | 64.3 | 160.7 | 180 | 8 | 60 | 61.3 |
| 59 | 270 | 180 | 760.5 | 972 | 70.7 | 176.8 | 180 | 8 | 60 | 54.38 |
| 60 | 270 | 180 | 760.5 | 972 | 77.1 | 192.9 | 180 | 8 | 60 | 49.83 |
| 61 | 180 | 180 | 774 | 1090.8 | 46.3 | 115.7 | 144 | 8 | 60 | 61.96 |
| 62 | 180 | 180 | 774 | 1090.8 | 51.4 | 128.6 | 144 | 8 | 60 | 65.26 |
| 63 | 180 | 180 | 774 | 1090.8 | 56.6 | 141.4 | 144 | 8 | 60 | 53.39 |
| 64 | 180 | 180 | 774 | 1090.8 | 61.7 | 154.3 | 144 | 8 | 60 | 52.4 |
| 65 | 210 | 210 | 810.6 | 966 | 54 | 135 | 168 | 8 | 60 | 57.54 |
| 66 | 210 | 210 | 810.6 | 966 | 60 | 150 | 168 | 8 | 60 | 64.28 |
| 67 | 210 | 210 | 810.6 | 966 | 66 | 165 | 168 | 8 | 60 | 50.64 |
| 68 | 210 | 210 | 810.6 | 966 | 72 | 180 | 168 | 8 | 60 | 49.44 |
| 69 | 225 | 225 | 760.5 | 972 | 57.9 | 144.6 | 180 | 8 | 60 | 54.97 |
| 70 | 225 | 225 | 760.5 | 972 | 64.3 | 160.7 | 180 | 8 | 60 | 61.9 |
| 71 | 225 | 225 | 760.5 | 972 | 70.7 | 176.8 | 180 | 8 | 60 | 54.38 |
| 72 | 225 | 225 | 760.5 | 972 | 77.1 | 192.9 | 180 | 8 | 60 | 53.79 |
| 73 | 293 | 88 | 760 | 1005 | 143.3 | 71.7 | 0 | 6 | 20 | 37.4 |
| 74 | 293 | 88 | 760 | 1005 | 107.5 | 107.5 | 0 | 6 | 20 | 26.6 |
| 75 | 293 | 88 | 760 | 1005 | 143.3 | 71.7 | 0 | 4 | 20 | 29.9 |
| 76 | 293 | 88 | 760 | 1005 | 107.5 | 107.5 | 0 | 4 | 20 | 30.7 |
| 77 | 344 | 34 | 760 | 1005 | 143.3 | 71.7 | 0 | 4 | 20 | 15.5 |
| 78 | 316 | 63 | 760 | 1005 | 143.3 | 71.7 | 0 | 4 | 20 | 23 |
| 79 | 293 | 88 | 760 | 1005 | 143.3 | 71.7 | 0 | 4 | 20 | 30.6 |
| 80 | 253 | 108 | 760 | 1005 | 143.3 | 71.7 | 0 | 4 | 20 | 39.01 |
| 81 | 272 | 108 | 760 | 1005 | 143.3 | 71.7 | 0 | 4 | 20 | 27.8 |
| 82 | 253 | 126 | 760 | 1005 | 143.3 | 71.7 | 0 | 4 | 20 | 28 |
| 83 | 237 | 158 | 547 | 1277 | 52 | 129 | 7.9 | 8 | 32 | 28.36 |
| 84 | 237 | 158 | 547 | 1277 | 52 | 129 | 7.9 | 4 | 32 | 34.84 |
| 85 | 237 | 158 | 547 | 1277 | 52 | 129 | 7.9 | 4 | 32 | 37.24 |
| 86 | 237 | 158 | 547 | 1277 | 52 | 129 | 7.9 | 4 | 32 | 33.2 |
| 87 | 400 | 0 | 651 | 1209 | 45.7 | 114.3 | 0 | 14 | 23 | 25.6 |
| 88 | 360 | 40 | 651 | 1209 | 45.7 | 114.3 | 0 | 14 | 23 | 38.3 |
| 89 | 340 | 60 | 651 | 1209 | 45.7 | 114.3 | 0 | 14 | 23 | 46.6 |
| 90 | 400 | 0 | 655.9 | 1218.1 | 40 | 100 | 6 | 14 | 23 | 32.5 |
| 91 | 360 | 40 | 655.9 | 1218.1 | 40 | 100 | 6 | 14 | 23 | 33.3 |
| 92 | 200 | 200 | 716 | 1074 | 9 | 56 | 0 | 0 | 0 | 31.7 |
| 93 | 200 | 200 | 712 | 1068 | 12 | 74 | 0 | 0 | 0 | 49.3 |
| 94 | 200 | 200 | 708 | 1062 | 15 | 93 | 0 | 0 | 0 | 58.2 |
| 95 | 200 | 200 | 712 | 1068 | 19 | 62 | 0 | 0 | 0 | 44.4 |
| 96 | 200 | 200 | 708 | 1062 | 15 | 93 | 0 | 0 | 0 | 58.2 |
| 97 | 200 | 200 | 712 | 1068 | 12 | 74 | 0 | 0 | 0 | 49.3 |
| 98 | 100 | 300 | 712 | 1068 | 12 | 74 | 0 | 0 | 0 | 52.1 |
| 99 | 0 | 400 | 712 | 1068 | 12 | 74 | 0 | 0 | 0 | 63.1 |
| 100 | 200 | 200 | 716 | 1074 | 15 | 93 | 0 | 0 | 0 | 65.7 |
| 101 | 200 | 200 | 708 | 1062 | 15 | 93 | 0 | 0 | 0 | 58.2 |
| 102 | 200 | 200 | 700 | 1050 | 15 | 93 | 0 | 0 | 0 | 42.3 |
| 103 | 360 | 40 | 651 | 1209 | 45.7 | 114.3 | 0 | 14 | 22 | 40 |
| 104 | 320 | 80 | 651 | 1209 | 45.7 | 114.3 | 0 | 14 | 22 | 47 |
| 105 | 360 | 40 | 651 | 1209 | 64 | 96 | 0 | 14 | 22 | 43 |
| 106 | 320 | 80 | 651 | 1209 | 64 | 96 | 0 | 14 | 22 | 54 |
| 107 | 400 | 0 | 658 | 1222 | 40 | 100 | 6 | 14 | 22 | 25 |
| 108 | 360 | 40 | 655 | 1216 | 40 | 100 | 6 | 14 | 22 | 27 |
| 109 | 320 | 80 | 655 | 1216 | 40 | 100 | 6 | 14 | 22 | 35 |
| 110 | 400 | 0 | 658 | 1222 | 56 | 84 | 6 | 14 | 22 | 27 |
| 111 | 360 | 40 | 655 | 1216 | 56 | 84 | 6 | 14 | 22 | 27 |
| 112 | 320 | 80 | 655 | 1216 | 56 | 84 | 6 | 14 | 22 | 45 |
| 113 | 360 | 40 | 644 | 1197 | 53 | 107 | 4 | 10 | 22 | 21.9 |
| 114 | 340 | 60 | 646 | 1200 | 53 | 107 | 4 | 10 | 22 | 28 |
| 115 | 320 | 80 | 648 | 1203 | 53 | 107 | 4 | 10 | 22 | 41.5 |
| 116 | 300 | 100 | 650 | 1207 | 53 | 107 | 4 | 10 | 22 | 46 |
| 117 | 280 | 120 | 652 | 1210 | 53 | 107 | 4 | 10 | 22 | 56.5 |
| 118 | 340 | 60 | 646 | 1200 | 53 | 107 | 4 | 12 | 22 | 35 |
| 119 | 320 | 80 | 648 | 1203 | 53 | 107 | 4 | 12 | 22 | 45 |
| 120 | 300 | 100 | 658 | 1222 | 53 | 107 | 4 | 12 | 22 | 57 |
| 121 | 340 | 60 | 651 | 1209 | 47 | 93 | 4 | 10 | 22 | 30 |
| 122 | 320 | 80 | 661 | 1227 | 47 | 93 | 4 | 10 | 22 | 40 |
| 123 | 300 | 100 | 671 | 1246 | 47 | 93 | 4 | 10 | 22 | 48 |
| 124 | 340 | 60 | 637 | 1184 | 64 | 96 | 4 | 10 | 22 | 28.5 |
| 125 | 300 | 100 | 659 | 1223 | 46 | 114 | 4 | 10 | 22 | 45 |
| 126 | 303.75 | 101.25 | 683 | 1269 | 81 | 81 | 4.05 | 8 | 0 | 10.5 |
| 127 | 303.75 | 101.25 | 683 | 1269 | 81 | 81 | 4.05 | 10 | 0 | 13 |
| 128 | 303.75 | 101.25 | 683 | 1269 | 81 | 81 | 4.05 | 12 | 0 | 19 |
| 129 | 303.75 | 101.25 | 683 | 1269 | 81 | 81 | 4.05 | 14 | 0 | 22 |
| 130 | 303.75 | 101.25 | 683 | 1269 | 81 | 81 | 4.05 | 16 | 0 | 24 |
| 131 | 303.75 | 101.25 | 683 | 1269 | 108 | 54 | 4.05 | 14 | 0 | 22.4 |
| 132 | 303.75 | 101.25 | 683 | 1269 | 64.8 | 97.2 | 4.05 | 14 | 0 | 27 |
| 133 | 303.75 | 101.25 | 683 | 1269 | 54 | 108 | 4.05 | 14 | 0 | 33 |
| 134 | 303.75 | 101.25 | 683 | 1269 | 46.28 | 115.72 | 4.05 | 14 | 0 | 30 |
| 135 | 303.75 | 101.25 | 683 | 1269 | 40.5 | 121.5 | 4.05 | 14 | 0 | 18.2 |
| 136 | 303.75 | 101.25 | 683 | 1269 | 81 | 81 | 4.05 | 8 | 0 | 22.2 |
| 137 | 303.75 | 101.25 | 683 | 1269 | 81 | 81 | 4.05 | 10 | 0 | 23.2 |
| 138 | 303.75 | 101.25 | 683 | 1269 | 81 | 81 | 4.05 | 12 | 0 | 24 |
| 139 | 303.75 | 101.25 | 683 | 1269 | 81 | 81 | 4.05 | 14 | 0 | 24.8 |
| 140 | 303.75 | 101.25 | 683 | 1269 | 81 | 81 | 4.05 | 16 | 0 | 21.8 |
| 141 | 303.75 | 101.25 | 683 | 1269 | 108 | 54 | 4.05 | 14 | 0 | 23.8 |
| 142 | 303.75 | 101.25 | 683 | 1269 | 64.8 | 97.2 | 4.05 | 14 | 0 | 33.8 |
| 143 | 303.75 | 101.25 | 683 | 1269 | 54 | 108 | 4.05 | 14 | 0 | 34.5 |
| 144 | 303.75 | 101.25 | 683 | 1269 | 46.28 | 115.72 | 4.05 | 14 | 0 | 35 |
| 145 | 303.75 | 101.25 | 683 | 1269 | 40.5 | 121.5 | 4.05 | 14 | 0 | 31.7 |
| 146 | 0 | 400 | 810 | 990 | 57.1 | 143 | 8 | 12 | 25 | 89.6 |
| 147 | 0 | 400 | 810 | 990 | 57.1 | 143 | 12 | 12 | 25 | 89.2 |
| 148 | 0 | 400 | 810 | 990 | 57.1 | 143 | 16 | 12 | 25 | 88.7 |
| 149 | 0 | 400 | 810 | 990 | 57.1 | 143 | 20 | 12 | 25 | 86.11 |
| 150 | 0 | 400 | 810 | 990 | 57.1 | 143 | 24 | 12 | 25 | 84.1 |
| 151 | 204.5 | 204.5 | 554 | 1293 | 41 | 102 | 0 | 10 | 0 | 53.5 |
| 152 | 102 | 307 | 554 | 1293 | 41 | 102 | 0 | 10 | 0 | 55.5 |
| 153 | 0 | 409 | 554 | 1293 | 41 | 102 | 0 | 10 | 0 | 58.6 |
| 154 | 0 | 400 | 740 | 1110 | 12.9 | 82.5 | 0 | 0 | 22 | 34.6 |
| 155 | 0 | 400 | 785 | 1085 | 12.9 | 82.5 | 0 | 0 | 22 | 53.6 |
| 156 | 0 | 400 | 790 | 1065 | 12.9 | 82.5 | 0 | 0 | 22 | 66.7 |
